# Hepatitis E Virus ORF1 Polyprotein Harbors a Pocket‐Like Cavity That Is Vital for Virus Replication and Represents a Novel Antiviral Target

**DOI:** 10.1002/advs.202501699

**Published:** 2025-10-02

**Authors:** Xiaohui Ding, Dou Zeng, Dan Liu, Yingying Bian, Bin Li, Zheng Li, Qiudi Li, Shiquan Liang, Yunlong Si, Qili Yao, Yibo Ding, Jiahui Zhu, Xiangyang Li, Kuiyang Zheng, Hongbo Guo, Wenshi Wang

**Affiliations:** ^1^ Department of Pathogen Biology and Immunology School of Basic Medical Sciences Xuzhou Medical University Xuzhou 221004 China; ^2^ Jiangsu Key Laboratory of Immunity and Metabolism Jiangsu International Laboratory of Immunity and Metabolism Xuzhou Medical University Xuzhou 221004 China; ^3^ Jiangsu Key Laboratory of Brain Disease and Bioinformation Research Center for Biochemistry and Molecular Biology Xuzhou Medical University Xuzhou 221004 China

**Keywords:** antiviral target, hepatitis E virus, ORF1 polyprotein, replication machinery, RNA‐dependent RNA polymerase, X domain

## Abstract

Hepatitis E virus (HEV) is the leading cause of acute viral hepatitis worldwide, yet no FDA‐approved anti‐HEV medication available. Elucidating HEV replication machinery is therefore crucial for identifying novel antiviral targets, and consequently developing potent antivirals. The nonstructural ORF1 polyprotein is pivotal for HEV replication. Herein, it is revealed that the ORF1 X domain is a critical component of HEV replication machinery. Interestingly, the ADP‐ribose hydrolase activity of X domain per se is dispensable for HEV replication. Instead, the X domain supports HEV replication through its interdomain interaction with the RNA‐dependent RNA polymerase (RdRp). Structure‐based functional analysis reveals that X and RdRp jointly create a “pocket‐like” cavity (PC) at their interaction interface. Site‐directed mutagenesis disrupting the PC integrity completely abolishes HEV replication, demonstrating its crucial role in the viral life cycle. Through a PC‐targeted high‐throughput in silico screening pipeline, combined with molecular docking analysis, surface plasmon resonance assays, and advanced in vitro HEV models, saikosaponin D and liriopesides B are identified as potent HEV inhibitors targeting this critical interface. Collectively, this study identifies a novel structure within ORF1 polyprotein that is crucial for HEV replication, and demonstrates the feasibility of developing novel antivirals by targeting this PC structure.

## Graphical abstract

Within the hepatitis E virus (HEV) ORF1 polyprotein, the X domain interacts with viral RNA‐dependent RNA polymerase (RdRp), forming a functional “pocket‐like” cavity (PC) that is crucial for viral replication. Structure‐based screening, surface plasmon resonance (SPR) assays, and HEV model validation collectively identify saikosaponin D and liriopesides B as PC‐targeting compounds with potent anti‐HEV activity, offering a novel therapeutic strategy.

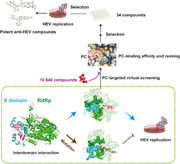

## 1 Introduction

Hepatitis E virus (HEV) is the leading cause of acute viral hepatitis and has emerged as a worldwide health challenge in both developing and industrialized countries. It leads to ≈ 20 million infections and 60 000 fatalities per annum.^[^ ^1^, ^2^ ^]^ Most HEV infections are self‐resolving or cause mild gastrointestinal symptoms (e.g., vomiting, abdominal pain, and nausea) in healthy individuals. However, it is easy to progress into chronic hepatitis, and even cirrhosis in immunosuppressed individuals.^[^ ^3^, ^4^ ^]^ Furthermore, HEV infection in patients with pre‐existing liver disease often leads to acute liver failure, and pregnant women face significantly higher mortality rates, reaching up to 30%.^[^ ^5^ ^]^ With the reported incidence in developed countries/regions (e.g., Europe) and the periodic outbreaks in resource‐limited countries/regions (e.g., South Sudan, Somalia, Uganda, and Chad) increasing, concerns about hepatitis E are tremendously growing. Despite this burden, treatment options remain limited to supportive care or off‐label ribavirin or pegylated interferon‐alpha (IFN‐α), with no proven anti‐HEV medication available.^[^ ^6^, ^7^, ^8^ ^]^ This critical gap underscores the urgent need to conduct a comprehensive investigation of HEV replication machinery to identify virus‐specific antiviral targets and develop potent and specific antivirals against HEV.

HEV is a positive‐sense, single‐stranded RNA (+ssRNA) virus classified within the *Hepeviridae* family and the *Orthohepevirus* genus.^[^ ^9^ ^]^ The HEV genome is ≈ 7.2 kb in length and consists of three open reading frames (ORFs). The viral capsid protein is encoded by ORF2,^[^ ^10^ ^]^ and ORF3 encodes a small, multifunctional protein that is required for the release of infectious virus particles.^[^ ^11^ ^]^ ORF1 encodes a nonstructural polyprotein that is pivotal for viral replication.^[^ ^12^ ^]^ The ORF1 polyprotein contains several functional domains, including a methyltransferase (Met), a Y domain, a papain‐like cysteine protease (PCP), a hypervariable region (HVR), an X domain, an RNA helicase (Hel), and an RNA‐dependent RNA polymerase (RdRp).^[^ ^13^ ^]^

X domain (also termed macro domain) is an evolutionarily ancient and highly conserved globular protein domain.^[^ ^14^, ^15^ ^]^ Encoded by multiple +ssRNA viruses across the *Togaviridae*, *Coronaviridae*, and *Hepeviridae* families, X domain exhibits ADP‐ribose hydrolase activity.^[^ ^16^, ^17^, ^18^ ^]^ It specifically counteracts poly(ADP‐ribose) polymerase (PARP)‐mediated ADP‐ribosylation, establishing the X domain as a key regulator of viral replication and pathogenesis.^[^ ^19^, ^20^, ^21^, ^22^ ^]^ However, the precise function of the X domain in the HEV life cycle remains poorly understood. This knowledge gap has impeded both the comprehensive characterization of ORF1 polyprotein in HEV replication and the identification of virus‐specific antiviral targets.

Herein, we demonstrated that the X domain of ORF1 polyprotein is a critical component of HEV replication machinery. Interestingly, the ADP‐ribose hydrolase activity of X domain per se is dispensable for HEV replication. Instead, X domain facilitates HEV replication through its intricate interdomain interaction with the RdRp domain. Structure‐based functional analysis revealed that the interaction between X and RdRp domains forms a “pocket‐like” cavity (PC) at their interdomain interface. Mutations of key PC‐forming residues (X domain: K937, Y943, R944; RdRp: G1590) completely abolished HEV replication, validating the PC's essential role. Using a PC‐targeted structure‐based high‐throughput virtual screening platform, we evaluated >10 000 commercially available compounds. Top hits with high predicted binding affinity to the PC underwent cell viability assays, and their anti‐HEV activity was assessed using advanced in vitro HEV models. Ultimately, two PC‐binding compounds (saikosaponin D and liriopesides B) were identified as potent anti‐HEV inhibitors. These data highlight the indispensable role of the PC in HEV replication and validate its potential as a novel anti‐HEV target for the development of potent antivirals. Taken together, our study underlines that deciphering the functions of HEV ORF1 polyprotein is essential for the in‐depth understanding of HEV replication machinery, and represents a novel strategy to identify potent and specific anti‐HEV targets.

## 2 Results

### 2.1 The ADP‐Ribose Hydrolase Activity of X Domain is Dispensable for HEV Replication

**Figure 1:**
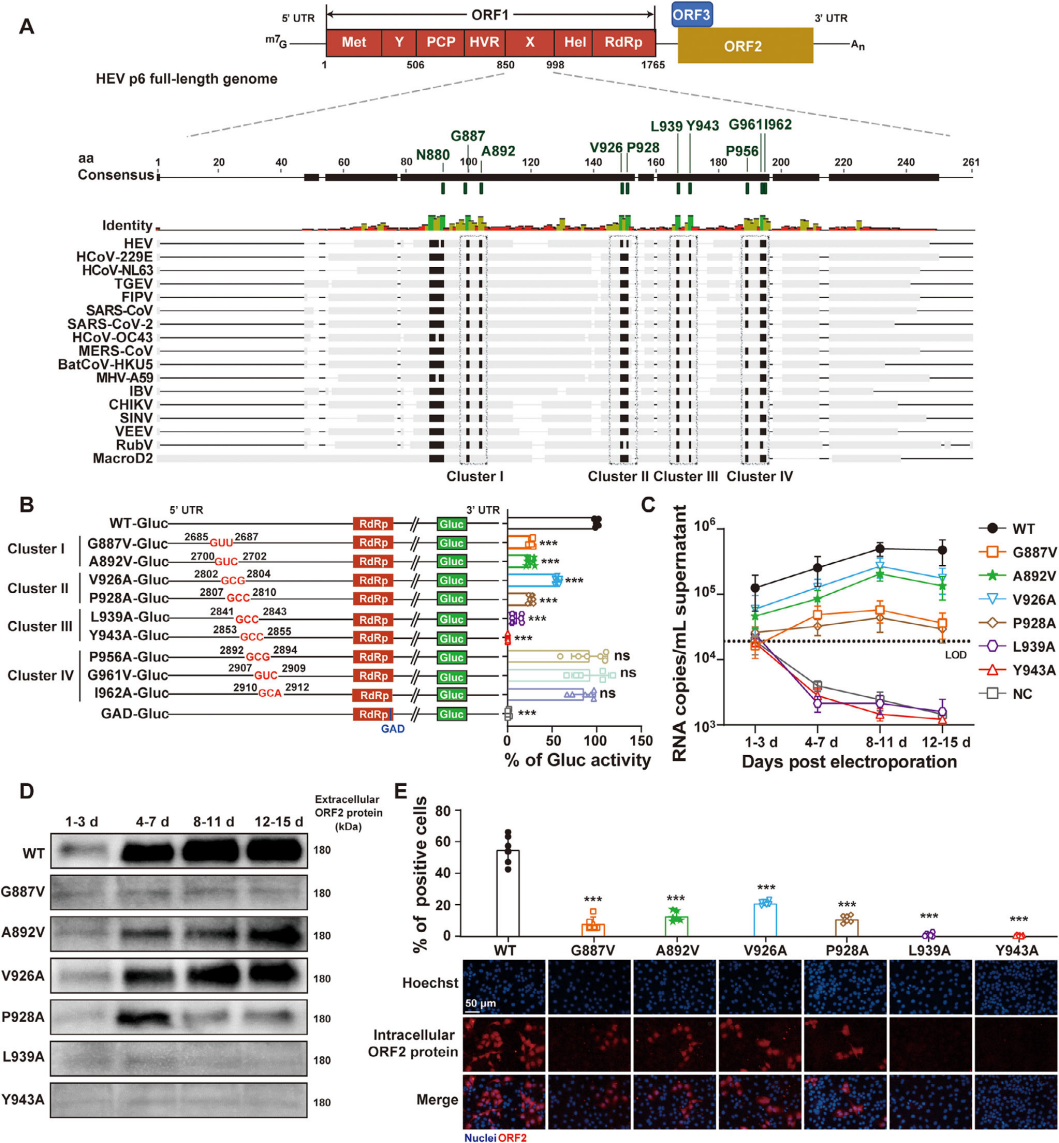
Mutating residues within Clusters I–III of X domain significantly impairs HEV replication. A) Sequence alignment of X domains across 16 +ssRNA viruses identified ten conserved residues in HEV, which were grouped into four clusters (I–IV), with N880 positioned outside these clusters. B) Site‐directed mutagenesis analysis of residues within Clusters I–IV of X domain in an HEV‐Gluc replicon model (Kernow‐C1/p6‐Gluc). Replicon mutant RNAs were electroporated into HuH7 cells, with replication‐defective GAD‐Gluc serving as the negative control. Viral replication efficiency was determined by measuring secreted Gluc activity at 48 h post‐electroporation. Data are presented as mean ± SD (*n* = 3), with wild‐type (WT) Gluc activity normalized to 100%. C–E) Site‐directed mutagenesis analysis of residues within Clusters I–III of X domain in a full‐length HEV infectious clone model (Kernow‐C1/p6). C) Extracellular viral RNA levels were quantified by RT‐qPCR at indicated time points from days 1–15 (*n* = 3). Viral RNA copy numbers were calculated from a standard curve and expressed as copies per milliliter (mL) of supernatant. LOD, limit of detection. D) Extracellular ORF2 protein dimers (≈180 kDa) were detected by native Western blot at indicated time points (*n* = 3). E) ORF2‐positive cells were quantified by immunofluorescence assay (IFA) (*n* = 3). Data are presented as mean ± SD. ^***^, *p *< 0.001; ns, not significant.

Identifying functionally important residues is fundamental to understanding protein biological functions. Sequence conservation analysis helps pinpoint functionally crucial residues.^[^ ^23^ ^]^ We therefore retrieved and analyzed X domain amino acid sequences from representative +ssRNA viruses (belonging to *Togaviridae*, *Coronaviridae*, and *Hepeviridae* families). In total, ten highly conserved residues were identified and mapped to the HEV X domain (**Figure** **1**A). Additional sequence analysis revealed that these residues were highly conserved across all eight HEV genotypes (Figure S1, Supporting Information). As expected, N880, the key residue for the X domain's ADP‐ribose hydrolase activity, was among the conserved residues. This prompted us to investigate whether this enzymatic activity is required for HEV replication. Previous studies show that the N880A mutation completely abolishes ADP‐ribose hydrolase activity.^[^ ^24^, ^25^, ^26^ ^]^ We therefore introduced this mutation into the HEV‐Gluc replicon (Kernow‐C1/p6‐Gluc), where the 5′ portion of HEV ORF2 is replaced with an in‐frame secreted *Gaussia* luciferase (Gluc)^[^ ^27^ ^]^ (Figure S2A, Supporting Information). Strikingly, the N880A‐Gluc mutant exhibited replication capacity comparable to that of the wild type (WT‐Gluc) (Figure S2B, Supporting Information), implying a dispensable role of the ADP‐ribose hydrolase activity in HEV replication. Consistently, a full‐length HEV infectious clone (Kernow‐C1/p6) carrying the N880A mutation exhibited replication kinetics comparable to the wild‐type clone (Figure S2C–G, Supporting Information). These results demonstrate that the ADP‐ribose hydrolase activity of the X domain is dispensable for HEV replication.

### 2.2 Mutation of Each Conserved Residue in Clusters I–III of the X Domain Causes Differential Attenuation of HEV Replication

Based on their spatial distribution within the X domain, the remaining nine conserved residues were classified into four clusters: Cluster I (G887, A892), Cluster II (V926, P928), Cluster III (L939, Y943), and Cluster IV (P956, G961, and I962) (Figure 1A). To investigate their functional significance in HEV replication, we generated individual point mutations at each target residue using the HEV‐Gluc replicon model (Figure 1B). A replication‐deficient construct (GAD‐Gluc) with a lethal mutation in the HEV RdRp domain served as the negative control.^[^ ^12^ ^]^ Notably, mutations in residues of Clusters I–III significantly impaired HEV replication. The V926A mutation reduced replication by ≈50%, while the G887V, A892V, P928A, and L939A mutations caused 80% to 90% reductions. Strikingly, the Y943A mutation completely abolished HEV replication. In contrast, mutations in residues of Cluster IV (P956A, G961V, and I962A) exerted no effect (Figure 1B).

We next introduced mutations of residues from Clusters I–III into the full‐length HEV infectious clone. Equal amounts of WT and mutant genomic RNAs, transcribed in vitro, were independently electroporated into HuH7 cells, and culture supernatants were then harvested at designated time points from days 1–15. Viral replication was assessed by quantifying HEV RNA (by RT‐qPCR) and ORF2 protein (by Western blot) in the supernatants. Consistent with the HEV‐Gluc replicon results, all six mutations attenuated viral replication to varying degrees (Figure 1C,D). Notably, the L939A and Y943A mutations completely blocked HEV replication. Consistently, intracellular ORF2 protein levels showed a similar pattern (Figure 1E).

### 2.3 Y943 of the X Domain is a Critical Residue for HEV Replication

**Figure 2:**
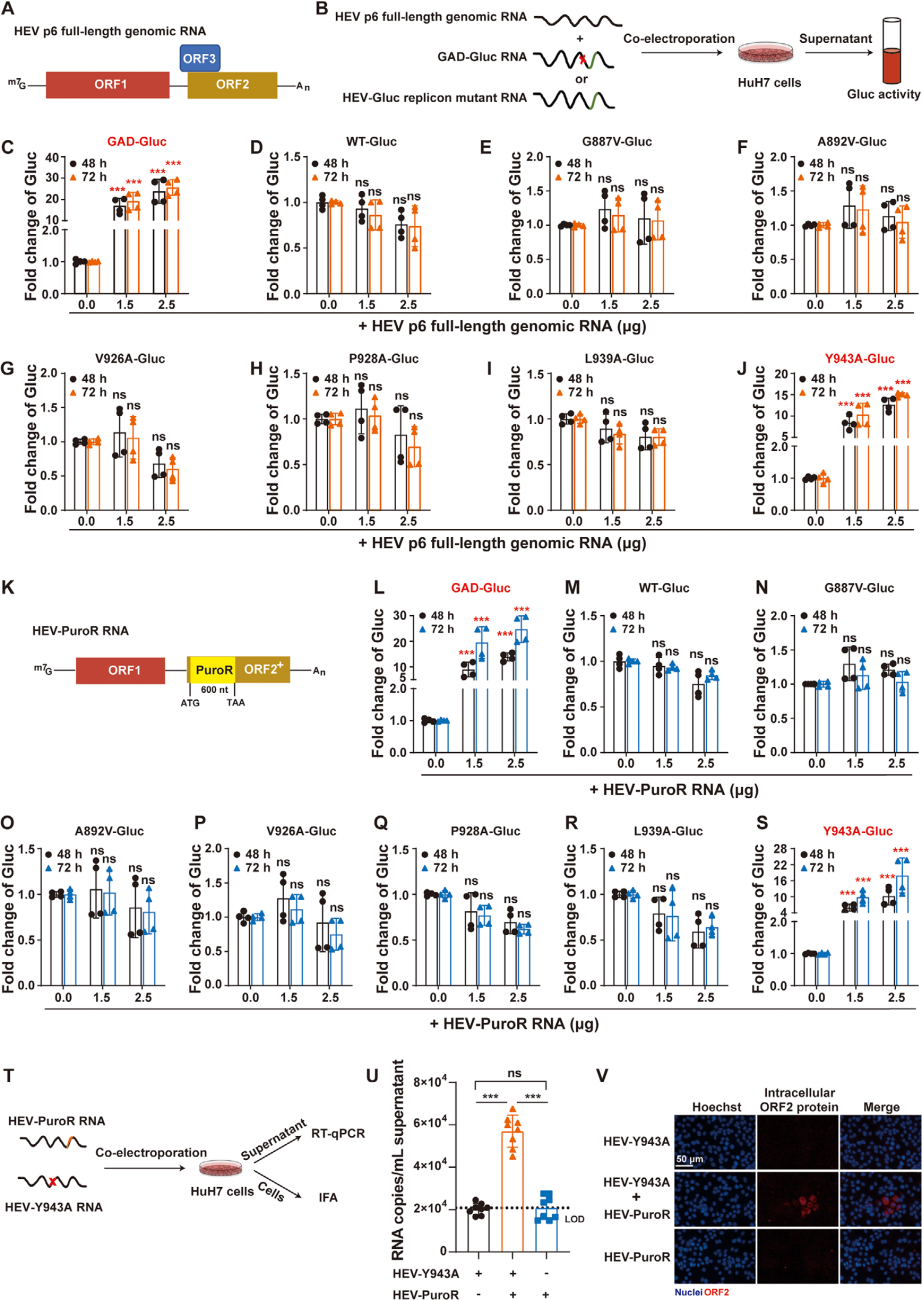
HEV Y943A mutant replication deficiency can be trans‐complemented by the exogenously expressed ORF1 polyprotein. A) Schematic of HEV p6 full‐length genomic RNA. B) Schematic of the HEV‐Gluc reporter system for trans‐complementation: HEV p6 full‐length genomic RNA supplies ORF1 polyprotein to rescue replication‐deficient mutants. C–J) HEV‐Gluc replicon mutant RNAs were co‐electroporated with HEV p6 full‐length genomic RNA, respectively, and Gluc activity was measured at 48  and 72 h (*n* = 4). K) Schematic of HEV‐PuroR genomic RNA. L–S) HEV‐Gluc replicon mutant RNAs were co‐electroporated with HEV‐PuroR RNA, respectively, and Gluc activity was measured at 48  and 72 h (*n* = 4). T–V) The full‐length HEV Y943A mutant RNA was co‐electroporated with HEV‐PuroR RNA. Extracellular HEV RNA levels at 72 h were quantified by RT‐qPCR (U), with viral RNA copy numbers calculated from a standard curve (copies mL^−1^ supernatant; n = 4; LOD, limit of detection). ORF2‐positive cells were detected by IFA (V). Data represent the mean ± SD. ***, *p *< 0.001; ns, not significant.

To elucidate the mechanisms underlying the functional impairments of these mutants, we established an HEV trans‐complementation Gluc reporter system by co‐electroporating GAD‐Gluc RNA^[^ ^12^ ^]^ and HEV p6 full‐length genomic RNA into HuH7 cells (**Figure** **2**A,B). In this system, ORF1 polyprotein expressed by HEV p6 full‐length genomic RNA trans‐complements GAD‐Gluc RNA, rescuing the replication‐deficient HEV‐Gluc replicon mutant as measured by Gluc activity (Figure 2C). Control experiments using WT‐Gluc RNA showed no additional Gluc activity enhancement (Figure 2D), confirming system specificity. Consequently, six HEV‐Gluc replicon mutant RNAs were individually co‐electroporated with HEV p6 full‐length genomic RNA. Gluc activity was measured at 48  and 72 h post‐electroporation. Notably, only Y943A‐Gluc was successfully rescued, while the other five mutants remained replication‐defective (Figure 2E–J). This observation is somewhat expected because site‐directed mutagenesis of ORF‐encoded residues inherently alters the corresponding nucleotide sequence. Consequently, the viral RNA secondary structures buried in this region might be negatively affected. Indeed, RNAalifold^[^ ^28^ ^]^ showed that the G887V, A892V, V926A, P928A, and L939A substitutions all introduced additional RNA loop structures in the X domain coding sequence (Figure S3, Supporting Information), likely contributing to the observed viral replication defects.

To further exclude potential confounding effects from ORF2 and ORF3 proteins expressed by HEV p6 full‐length genomic RNA, we engineered an HEV‐PuroR construct by replacing the 5′ portion of HEV ORF2 with the in‐frame puromycin resistance gene (Figure 2K). Six HEV‐Gluc replicon mutant RNAs were then individually co‐electroporated with HEV‐PuroR RNA. Gluc activity was measured at 48  and 72 h post‐electroporation. Consistent with Figure 2E–J, only Y943A‐Gluc replication deficiency could be rescued *in trans* (Figure 2N–S), indicating that the Y943A mutation impaired the function of X domain at the protein level. This conclusion was further corroborated by the ability of HEV‐PuroR RNA to rescue the replication competence of the full‐length infectious clone Y943A mutant (HEV‐Y943A), as evidenced by both viral RNA quantification and ORF2 protein expression (Figure 2T–V). Collectively, the conserved Y943 of the X domain is a critical residue for HEV replication.

### 2.4 The X Domain Functions as an Indispensable Subunit of the Intact ORF1 Polyprotein to Support HEV Replication

**Figure 3:**
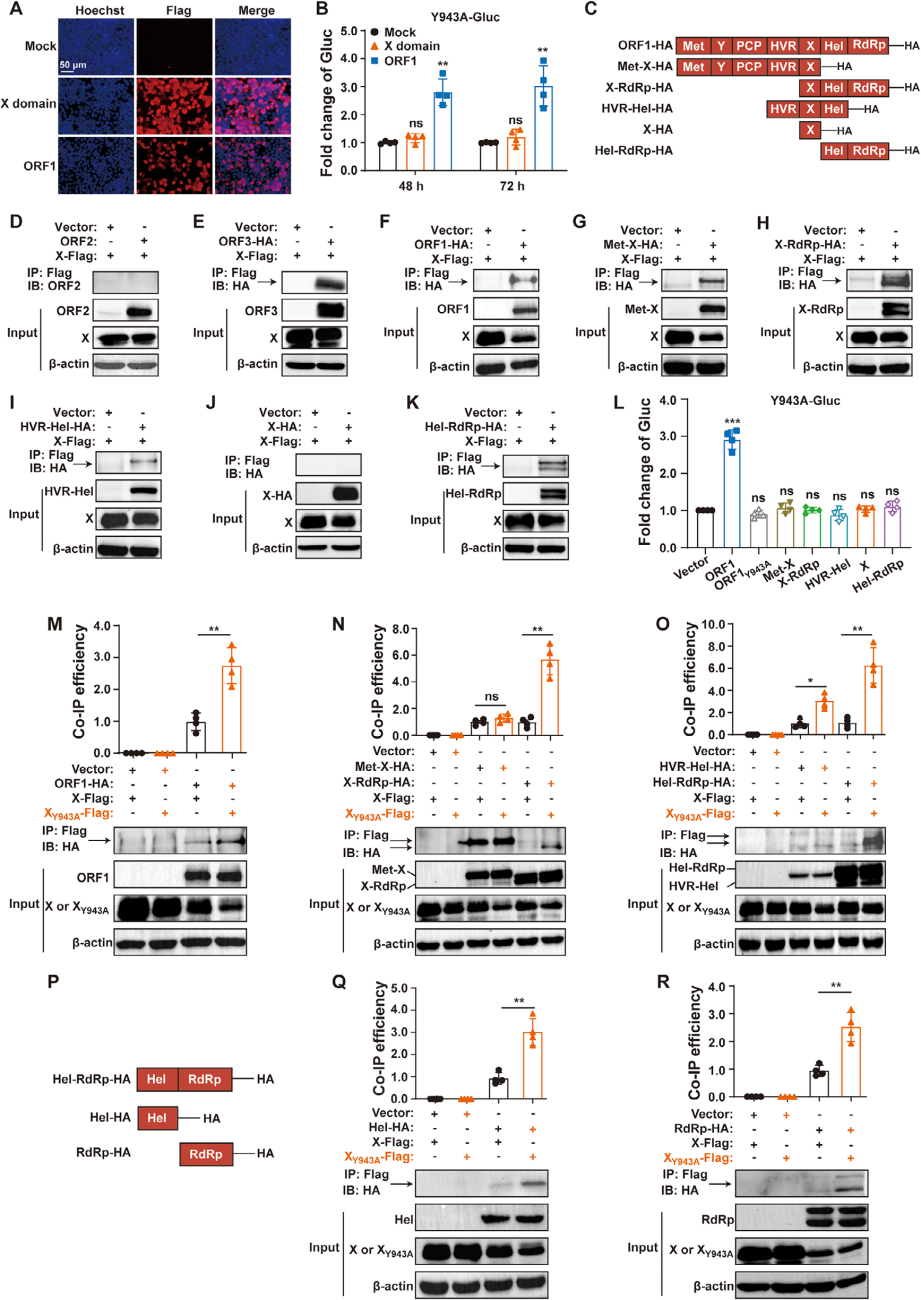
The X domain functions as an indispensable subunit of the intact ORF1 polyprotein, where the Y943A mutation strengthens its interdomain interactions with both Hel and RdRp. A,B) Y943A‐Gluc RNA was electroporated into HEK293T cells overexpressing either mock control, X domain, or the full‐length ORF1 polyprotein (A). Gluc activity was measured at 48  and 72 h (B, *n* = 4). C–K) A series of plasmid constructs encoding HA‐tagged truncated ORF1 variants were co‐transfected with the plasmid expressing C‐terminal Flag‐tagged X domain into HEK293T cells, respectively. Protein‐protein interactions were assessed by Co‐IP assays, with HEV ORF2 and ORF3 proteins serving as negative and positive controls, respectively. L) HEK293T cells overexpressing the truncated ORF1 variant were electroporated with Y943A‐Gluc RNA. Gluc activity was measured at 48 h post‐electroporation (*n* = 4). M–R) HEK293T cells were co‐transfected with plasmids encoding HA‐tagged truncated ORF1 variant and either Flag‐tagged X domain or Flag‐tagged X_Y943A_ domain mutant. Protein‐protein interactions were assessed by Co‐IP assays, with binding efficiency quantified using ImageJ grayscale analysis (*n* = 4). Data represent the mean ± SD. ^*^, *p *< 0.05; ^**^, *p *< 0.01; ^***^, *p *< 0.001; ns, not significant.

Since the Y943A mutant replication deficiency could be trans‐complemented by exogenously expressed ORF1 polyprotein, we next investigated whether the isolated X domain alone could similarly rescue its replication competence. To this end, we electroporated Y943A‐Gluc RNA into HEK293T cells overexpressing either mock control, isolated X domain, or full‐length ORF1 (**Figure** **3**A). Consistent with Figure 2, the full‐length ORF1 polyprotein successfully rescued Y943A‐Gluc replication. Nonetheless, the isolated X domain exhibited no trans‐complementation activity (Figure 3B). These results indicate that the X domain may not function autonomously, but rather serves as an indispensable part of a larger multi‐domain protein complex. To identify which domains are associated with the X domain and how they function together as an intact polyprotein, we generated a panel of constructs expressing either the X domain alone or combined with adjacent domains (Figure 3C). Co‐IP assays revealed that the X domain could interact with the full‐length ORF1 polyprotein, as well as its truncated variants, including Met‐X, X‐RdRp, HVR‐Hel, and Hel‐RdRp (Figure 3F–K). Following established validation protocols,^[^ ^29^ ^]^ we employed HEV proteins ORF2 and ORF3 as negative and positive controls to ensure the reliability of our assay (Figure 3D,E). Next, HEK293T cells were transfected with each of these constructs, followed by electroporation of Y943A‐Gluc RNA. The wild‐type ORF1, but not the ORF1_Y943A_ mutant, rescued Y943A‐Gluc replication competence. Strikingly, none of the truncated ORF1 variants, irrespective of X domain presence, could complement Y943A‐Gluc replication deficiency (Figure 3L). Taken together, these results demonstrate that instead of operating as an autonomous functional unit, X domain functions as an indispensable subunit of the intact ORF1 polyprotein to support HEV replication.

### 2.5 The Y943A Mutation Enhances X Domain Interactions with Both Hel and RdRp

To explore the impact of Y943A mutation on the interactions of X domain with other ORF1 regions, X domain carrying Y943A mutation (designated X_Y943A_ domain) and its wild type were subjected to protein‐protein interaction analyses, respectively. Strikingly, X_Y943A_ displayed stronger binding ability to the full‐length ORF1 polyprotein than its wild type (Figure 3M). While Y943A mutation had no detectable effect on the interaction of X domain with Met‐X, it significantly strengthened the binding of X domain to X‐RdRp, HVR‐Hel, and Hel‐RdRp fragments (Figure 3N,O). Notably, all these truncated variants contain either Hel or RdRp domain. Therefore, two constructs expressing HA‐tagged Hel and RdRp were generated and subjected to Co‐IP assays (Figure 3P). Importantly, X_Y943A_ exhibited markedly stronger interactions with both Hel and RdRp than its wild type (Figure 3Q,R).

**Figure 4:**
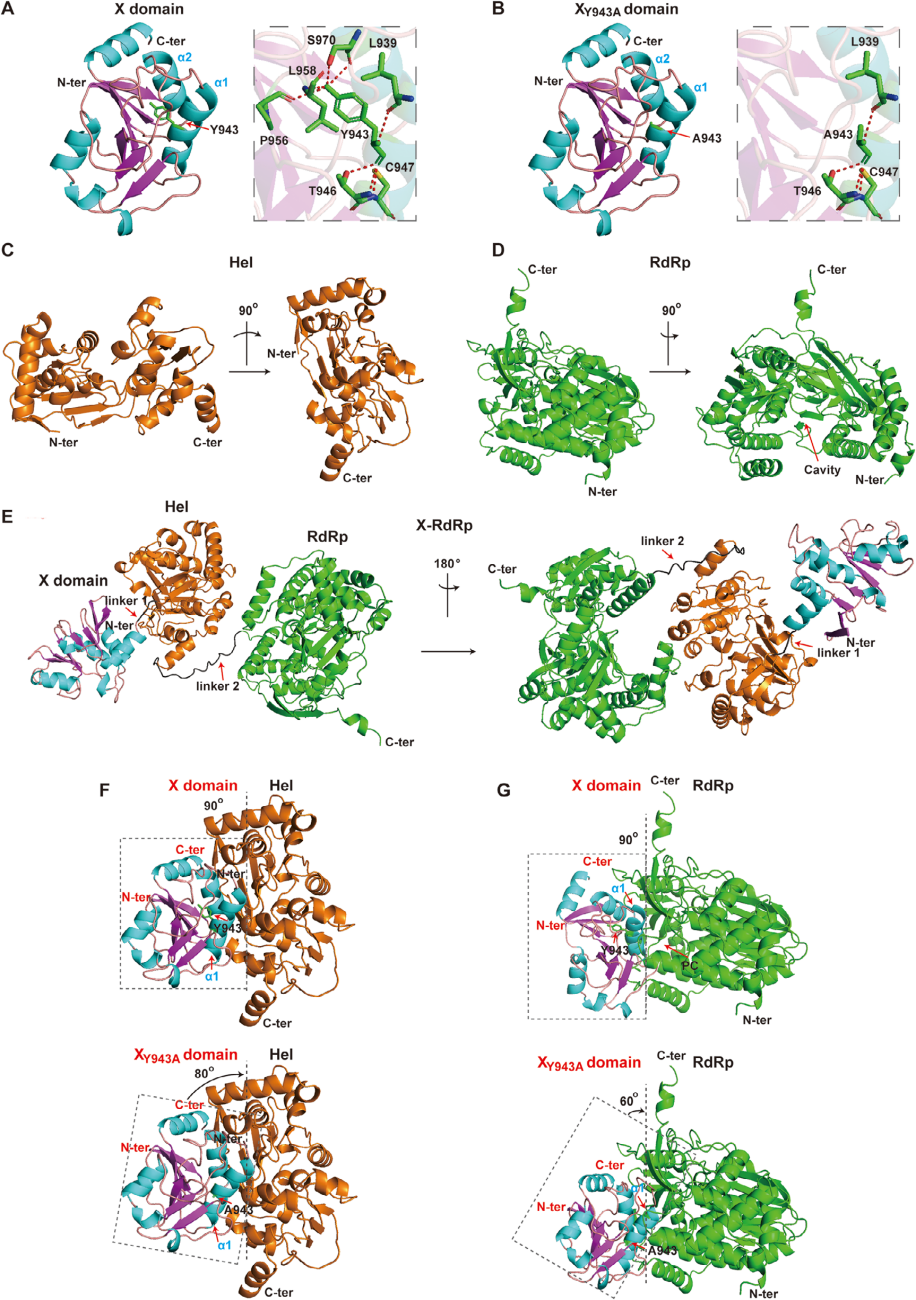
Structural basis of strengthened interdomain interactions of X_Y943A_ domain with both Hel and RdRp. A–E) The structural models of X (aa 850–998), X_Y943A_, Hel (aa 1002–1279), RdRp (aa 1298–1765), and X‐RdRp (aa 850–1765) domains were generated using Swiss Model, with the published well‐refined 3D structures of HEV ORF1 polyproteins (genotypes 1 and 3) as templates. F,G) Interdomain interactions of X domain (or X_Y943A_ domain) with both Hel and RdRp were analyzed using GRAMM‐based protein docking. Structures were visualized using PyMOL (v2.3.3).

These findings motivated us to investigate the structural basis underlying the strengthened interdomain interactions of X_Y943A_ with both Hel and RdRp domains. We utilized the published well‐refined 3D models of full‐length HEV ORF1 polyprotein (genotypes 1 and 3) as reference templates, which integrate both crystallographic structures and other generated domain models.^[^ ^30^, ^31^ ^]^ Using Swiss Model,^[^ ^32^, ^33^ ^]^ we generated structural models of X domain (aa 850–998), X_Y943A_, Hel (aa 1002–1279), RdRp (aa 1298–1765), and X‐RdRp (aa 850–1765) domains (**Figure** **4**A–E). Consistent with prior reports,^[^ ^30^, ^31^ ^]^ our analysis revealed that X domain adopted a structure comprising β sheets and internal loops sandwiched in by five α helices (Figure 4A). Y943, positioned in the α1 helix (^936^PKRLEAA**Y**RETCS^948^), formed hydrogen bonds with L939, T946, and C947 of α1 helix. More importantly, Y943 also formed hydrogen bonds with S970 of α2 helix (^967^VSLSFDAWER^976^), P956 and L958 of the internal loop (^956^PLLGSGIYQVP^966^, between α1 and α2 helix). These interactions collectively stabilized the 3D architecture of the X domain (Figure 4A, right). Notably, while Y943A mutation preserved the overall architecture of the α1 helix, it completely abolished hydrogen bonds between Y943 and residues in both the α2 helix and the internal loop (Figure 4B). Hel model exhibited a bi‐lobe core structure essential for helicase activity, which unwinds RNA duplex substrates at the interface between the two lobes (Figure 4C). RdRp model displayed the canonical “right‐hand” fold, comprising fingers, palm, and thumb (Figure 4D).

Interestingly, the X and Hel domains maintained close spatial proximity through a short 4‐residue linker (linker 1, Figure 4E), facilitating critical interdomain interaction. Indeed, Global Range Molecular Matching (GRAMM) server‐based protein docking analysis^[^ ^34^ ^]^ revealed that the α1 helix of X domain was located adjacent to the interdomain interface between the X and Hel domains (Figure 4F, top). Hydrogen bonds between Y943 and residues in both the α2 helix and the internal loop stabilized this interaction (Figure S4A, left, Supporting Information). However, the Y943A substitution disrupted this interaction network (Figure S4A, right, Supporting Information), destabilizing this interaction and causing X to tilt toward Hel (Figure 4F, bottom). In contrast, a flexible 20‐residue linker (linker 2, Figure 4E) between Hel and RdRp domains enabled significant RdRp repositioning, therefore facilitating interaction with the X domain. GRAMM server‐based protein docking analysis showed that α1 helix of X domain was located at the interaction interface between X and RdRp. However, the Y943A substitution eliminated hydrogen bonds between Y943 and residues in both the α2 helix and the internal loop, causing α1 helix to protrude toward the RdRp interface (Figure 4G; Figure S4B, Supporting Information). These structural rearrangements mechanistically explain the strengthened interdomain interactions of X_Y943A_ with both the Hel and RdRp.

### 2.6 Mutating Y943 of the X Domain Destroys the “Pocket‐Like” Cavity Located at the Interdomain Interface between X and RdRp and Abolishes HEV Replication

**Figure 5:**
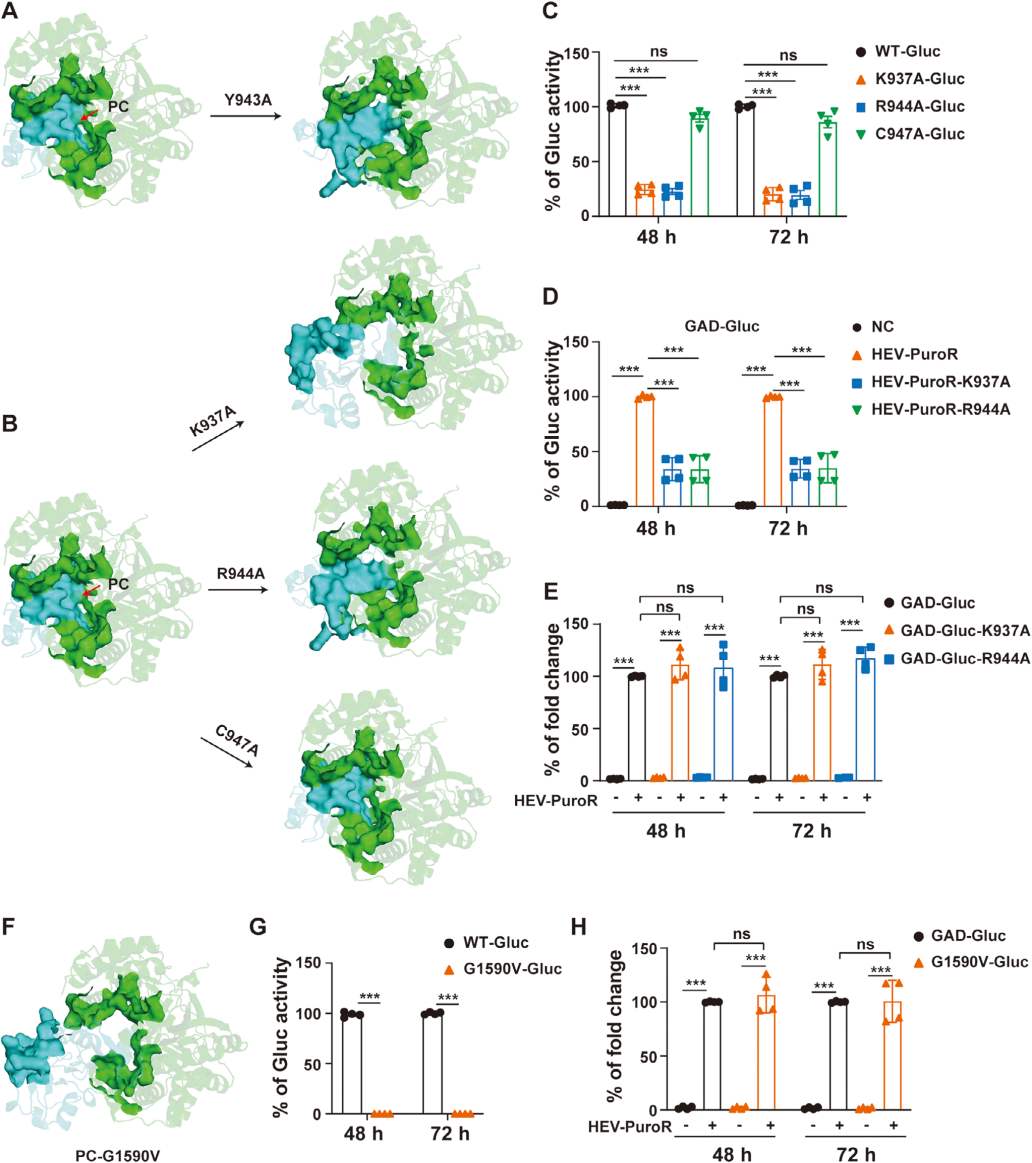
The structural integrity of the PC is essential for HEV replication. A) The Y943A substitution significantly disrupted the PC structure. B) K937A and R944A substitutions within the X domain severely destabilized the PC structure, whereas the C947A had no significant structural impact. C) Effect of K937A and R944A mutations on HEV replication in the HEV‐Gluc replicon model, with the C947A serving as a control. At 48  and 72 h post‐electroporation, Gluc activity was measured (*n* = 4), with WT‐Gluc normalized to 100%. D) HEV‐PuroR RNA carrying K937A or R944A mutation showed significantly impaired complementation efficiency in GAD‐Gluc trans‐complementation assays (*n* = 4), compared to wild‐type HEV‐PuroR RNA (normalized to 100%). E) HEV‐PuroR RNA successfully rescued replication of GAD‐Gluc carrying K937A or R944A mutation (*n* = 4), with wild‐type GAD‐Gluc complementation efficiency normalized to 100%. F) The G1590V substitution within RdRp domain strongly disturbed the PC structure. G) The G1590V mutation completely abolished HEV replication in the HEV‐Gluc replicon model (*n* = 4). H) HEV‐PuroR RNA could rescue G1590V‐Gluc replication activity, with GAD‐Gluc as a positive control (*n* = 4). Data represent the mean ± SD. ^***^, *p *< 0.001; ns, not significant.

Notably, regarding the interaction between the X and RdRp domains, the α1 helix of the X domain was positioned at one end of the RdRp cavity, forming a complete “pocket‐like” cavity (PC) (Figure 4D,G). CurPocket structure‐based cavity detection analysis^[^ ^35^ ^]^ identified this PC as the C1 pocket (volume 3428 Å^3^; cavity size 21, 24, and 30), formed by residues from both domains (highlighted in red, Figure S5A, Supporting Information). Residues K937, R938, A941, R944, E945, C947, and S948 within the X domain's α1 helix constituted the PC base (Figure S5B, Supporting Information). While Y943 did not directly contribute to the PC formation, the Y943A substitution disrupted its hydrogen bonds with S970, P956, and L958, resulting in α1 helix displacement (Figure 4G, bottom) and PC collapse (**Figure** **5**A).

The complete replication blockade caused by the Y943A mutation indicated the PC's essential role in HEV replication. To validate this finding, we introduced mutations into three PC base‐forming residues (K937, R944, and C947) within the X domain in HEV‐Gluc replicon (Figure S5B, Supporting Information). The K937A and R944A mutations severely disrupted the PC architecture, whereas the C947A exerted no structural effect (Figure 5B), thus serving as an optimal negative control. Correspondingly, the K937A and R944A mutations strongly inhibited HEV replication, whereas the C947A exhibited no effect (Figure 5C). In HEV trans‐complementation assays, HEV‐PuroR RNA carrying K937A or R944A failed to effectively rescue GAD‐Gluc replication, revealing ORF1 polyprotein dysfunction (Figure 5D). Conversely, wild‐type HEV‐PuroR RNA successfully rescued the replication of GAD‐Gluc RNA containing each mutation (Figure 5E). We extended this analysis to RdRp by mutating the PC‐forming residue G1590 (marked by #, Figure S5A, Supporting Information). G1590V mutation strongly disrupted the PC structure (Figure 5F). Like the K937A and R944A mutations, G1590V abolished HEV replication, a defect successfully rescued by wild‐type HEV‐PuroR RNA (Figure 5G,H). These results conclusively demonstrate that the PC located at the interdomain interface between the X and RdRp is vital for HEV replication, and any disruption of the PC structure causes lethal effect on HEV replication.

### 2.7 The PC Represents a Novel Antiviral Target for the Development of Antivirals against HEV

**Figure 6:**
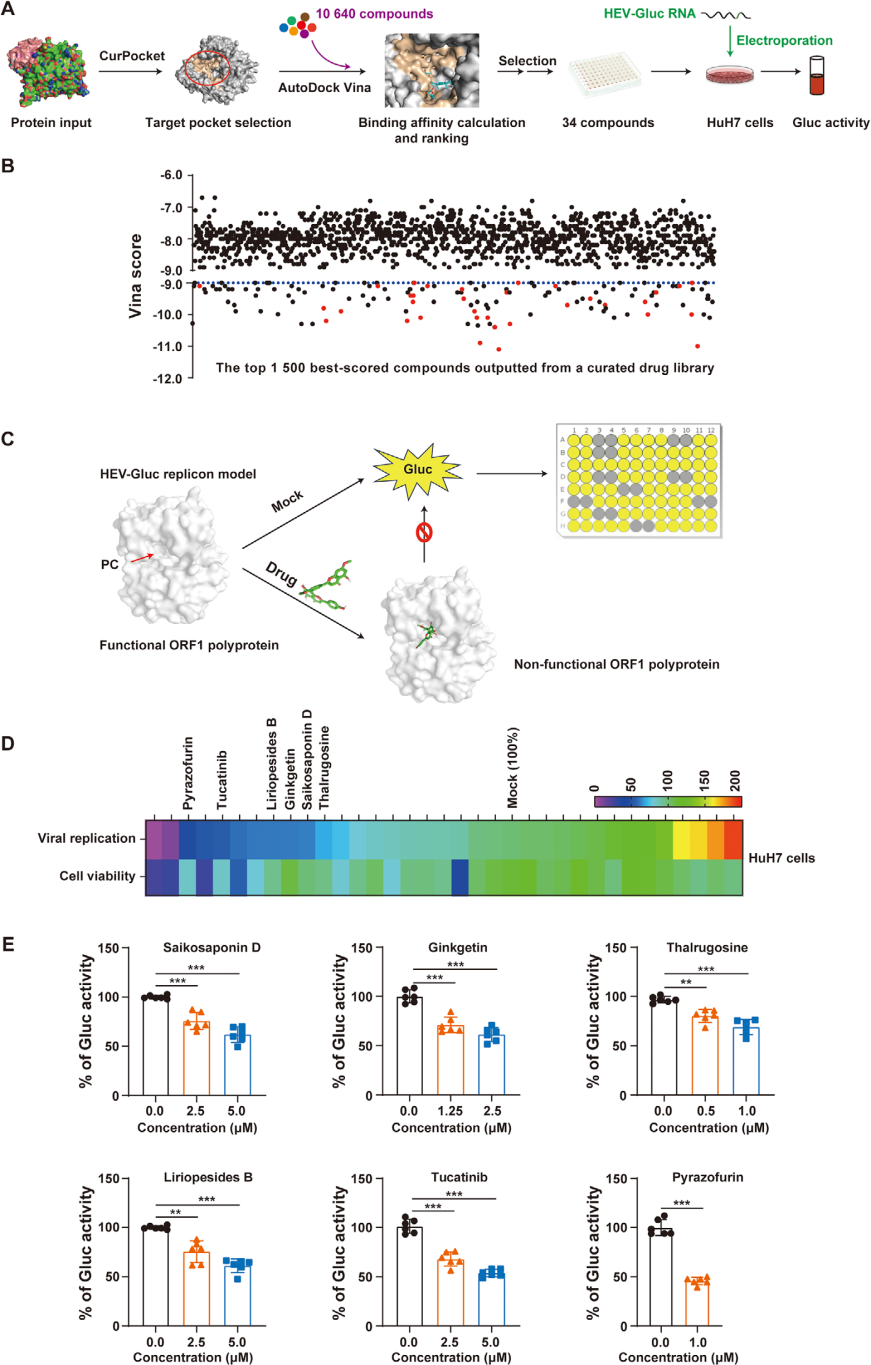
Identification of anti‐HEV compounds based on a PC‐targeted in silico drug screening and in vitro HEV‐Gluc replicon model. A,B) Drug screening workflow. Receptor‐based virtual screening was performed using DrugRep over a curated drug library (10 640 compounds).^[^ ^36^ ^]^ The top 1 500 compounds (the maximum output from DrugRep) with high predicted binding affinity to the PC were selected (Datasets 1–3). Vina scores are ranging from −6.0 (weak binding affinity) to −12.0 (strong binding affinity). After applying selection criteria: (i) Vina score ≥‐9.0 and (ii) exclusion of structurally similar compounds (>70% similarity), 34 commercially available candidates (red dots) were prioritized for experimental validation in the HEV‐Gluc replicon model (B). C) Schematic of the HEV‐Gluc replicon model‐based drug screening platform. HuH7 cells electroporated with HEV‐Gluc replicon RNA were treated with test compounds at the indicated concentrations, followed by viral replication (Gluc activity) and cell viability assays at 72 h. D) Heatmap showed the effect of each compound on viral replication and cell viability. E) Five compounds, along with the positive control pyrazofurin, demonstrated HEV replication inhibition in the HEV‐Gluc replicon model, with Gluc activity normalized to untreated cells (100%; mean ± SD, *n* = 3). ^**^, *p *< 0.01; ^***^, *p *< 0.001.

**Figure 7:**
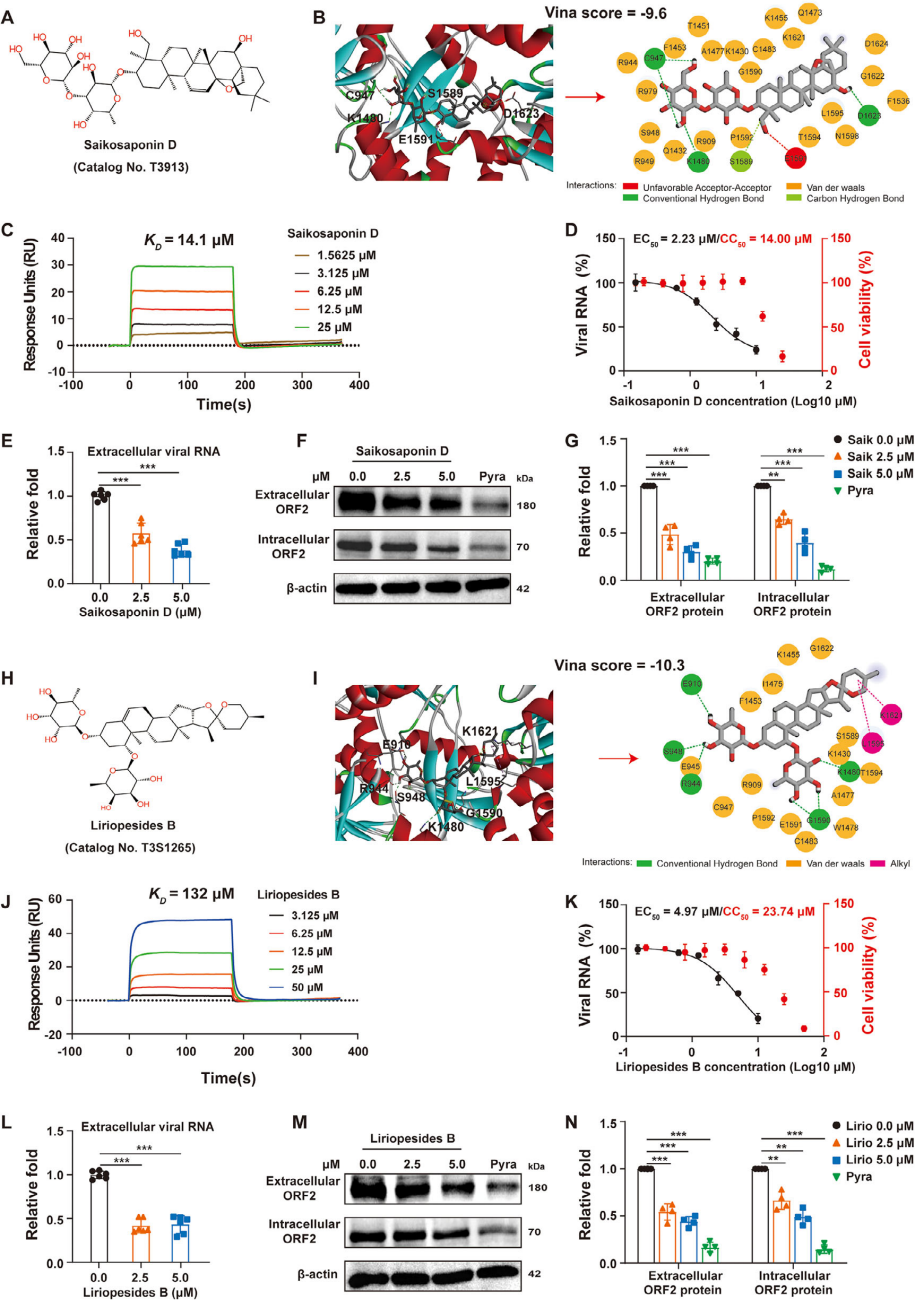
Saikosaponin D and liriopesides B are identified as potent anti‐HEV compounds targeting the PC. A–G) Antiviral activity of saikosaponin D in the full‐length HEV infectious clone model. A) Chemical structure of saikosaponin D. B) Molecular docking analysis of saikosaponin D with the PC using Discovery Studio Visualizer software. C) The binding affinity of saikosaponin D to the PC was determined by SPR assay. D–G) Anti‐HEV activity and cytotoxic effect of saikosaponin D. HEV RNA levels (extracellular and intracellular) were quantified by RT‐qPCR (*n* = 3), while HEV ORF2 protein levels (extracellular and intracellular) were detected by Western blot. Grayscale quantification of ORF2 in Western blot assays was determined by ImageJ (*n* = 4). H–N) Antiviral activity of liriopesides B in the full‐length HEV infectious clone model. H) Chemical structure of liriopesides B. I) Molecular docking analysis of liriopesides B with the PC using Discovery Studio Visualizer software. J) The binding affinity of liriopesides B to the PC was determined by SPR assay. K–N) Anti‐HEV activity and cytotoxic effect of liriopesides B. HEV RNA levels (extracellular and intracellular) were quantified by RT‐qPCR (*n* = 3), while HEV ORF2 protein levels (extracellular and intracellular) were detected by Western blot. Grayscale quantification of ORF2 in Western blot assays was determined by ImageJ (*n* = 4). EC_50_ was determined using intracellular HEV RNA quantification, while CC_50_ was evaluated in parallel by WST‐1 assay. Dose‐response curves were generated using nonlinear regression in GraphPad Prism version 9 (*n* = 3), with red dots indicating cytotoxicity. Pyra: pyrazofurin, positive control; Saik: saikosaponin D; Lirio: liriopesides B. Data are presented as mean ± SD. ^**^, *p *< 0.01; ^***^, *p *< 0.001.

Based on these findings, we identified the PC as a promising antiviral target. Therefore, we performed receptor‐based in silico virtual screening using DrugRep over a curated drug library (10 640 compounds)^[^ ^36^ ^]^ (**Figure** **6**A). From the top 1 500 compounds (the maximum output provided by DrugRep) with predicted high PC‐binding affinity (Figure 6B and Datasets 1–3), we applied the following selection criteria: i) Vina score was ≥‐9.0 and ii) exclusion of compounds with >70% structural similarity. This yielded 34 commercially available candidates (Table S1, Supporting Information), which were further evaluated for their anti‐HEV activity in the HEV‐Gluc replicon model (Figure 6C). Pyrazofurin, a known anti‐HEV agent, served as the positive control.^[^ ^27^ ^]^ At 72 h post‐treatment, viral replication and cell viability were assessed. Hits were defined as compounds that reduced Gluc activity by >40% with <20% cytotoxicity (Figure 6D; Table S2, Supporting Information). Herein, five compounds (saikosaponin D, ginkgetin, thalrugosine, liriopesides B, and tucatinib) showed potent HEV replication inhibition (Figure 6E; Figure S6, Supporting Information). Further validation in the full‐length HEV infectious clone model revealed that saikosaponin D and liriopesides B significantly reduced both extracellular and intracellular HEV RNA and ORF2 protein levels, whereas ginkgetin, thalrugosine, and tucatinib showed no significant inhibition (**Figure** **7**; Figure S7, Supporting Information).

Molecular docking analysis combined with surface plasmon resonance (SPR) quantification showed that both saikosaponin D and liriopesides B could bind to the PC in a dose‐dependent manner, with dissociation constants (*K_D_*) of 14.1 µm for saikosaponin D and 132 µm for liriopesides B (Figure 7A–C,H–J; Figures S8 and S9, Supporting Information). Consequently, both compounds exhibited potent anti‐HEV activity (Figure 7D–G,K–N). Specifically, the EC_50_ values of saikosaponin D and liriopesides B are 2.23 and 4.97 µm, respectively (Figure 7D,K). Moreover, oral gavage of saikosaponin D (at a single dose of 400 mg kg^−1^) achieved a time to maximum concentration (*T*_max_) of 1.00 h, with a maximum plasma concentration (*C*_max_) of 4.46 µg mL^−1^ (5.71 µm) (Figure S10A–D, Supporting Information). Similarly, oral gavage of liriopesides B (at a single dose of 400 mg kg^−1^) resulted in a *T*_max_ of 2.00 h, with a *C*_max_ of 3.68 µg mL^−1^ (5.09 µm) (Figure S10E–H, Supporting Information). Thus, saikosaponin D and liriopesides B exhibited binding affinity to the PC, potent anti‐HEV activity, and promising pharmacological potential. Taken together, the PC located at the interdomain interface between the X and RdRp domains serves as a novel antiviral target for the development of virus‐specific drugs against HEV.

## 3 Discussion

HEV RNA replication is driven by the viral nonstructural polyprotein ORF1.^[^ ^12^ ^]^ However, the functional roles of ORF1 domains have not been systematically elucidated, primarily due to a lack of comprehensive studies. This knowledge gap significantly hinders our understanding of their roles in the HEV replication cycle. In this study, we systematically investigated the unexplored X domain of ORF1. Protein function is primarily determined by molecular interactions at conserved sites or regions, where sequence conservation analysis can pinpoint the functionally crucial residues.^[^ ^23^ ^]^ Therefore, we performed sequence alignment of X domains from representative +ssRNA viruses (*Togaviridae*, *Coronaviridae*, and *Hepeviridae* families) and identified ten highly conserved residues in HEV. As expected, N880, the key residue for ADP‐ribose hydrolase activity of X domain, was among these conserved residues. X domain has been proved to be endowed with ADP‐ribose hydrolase activity in multiple +ssRNA viruses,^[^ ^16^, ^17^, ^18^ ^]^ which plays an important role in viral replication and pathogenesis by counteracting host antiviral ADP‐ribosylation.^[^ ^19^, ^20^, ^21^, ^22^ ^]^ Strikingly, the N880A mutation, which completely abolishes this enzymatic activity,^[^ ^24^, ^25^, ^26^ ^]^ exerted no detectable impact on HEV replication. This finding clearly demonstrates that the ADP‐ribose hydrolase activity per se is dispensable for HEV replication.

We next systematically examined the remaining nine conserved residues for their roles in HEV replication. Notably, mutations of residues within Clusters I–III significantly impaired viral replication, with L939A and Y943A completely abolishing HEV replication. Intriguingly, only the Y943A‐Gluc mutant could be rescued through the HEV trans‐complementation system, while the other five mutants remained replication‐defective. This phenotypic divergence is somewhat expected, likely reflecting the dual‐layered information encoded in viral RNA genomes.^[^ ^37^ ^]^ On one layer, the primary sequence directs the synthesis of viral proteins, which are essential for replication and virion production. The second layer of information is buried in the higher‐order RNA structures, formed through intramolecular base‐pairing and compaction.^[^ ^38^ ^]^ These viral RNA structures provide critical docking sites for the interaction of viral genomic RNA with viral proteins or host factors.^[^ ^39^ ^]^ RNA structure elements and their dynamic conformational switching are essential for the life cycles of diverse RNA viruses, with structural perturbations often disrupting viral replication. Our identification of five crucial residues (G887, A892, V926, P928, and L939) indicates the existence of essential RNA structural elements within the HEV genome. These findings highlight the need for single‐base resolution mapping of the full‐length HEV genome secondary structure, which would be invaluable for developing RNA‐targeted therapeutics against HEV infection.^[^ ^40^, ^41^ ^]^

Our data showed that only the full‐length ORF1 polyprotein, but not its truncated variants (irrespective of the X domain inclusion), rescued the Y943A mutant replication defect, establishing the X domain as an indispensable structural and functional component of the intact ORF1 polyprotein. Although proteolytic processing of ORF1 during HEV life cycle remains controversial,^[^ ^42^, ^43^, ^44^, ^45^, ^46^, ^47^ ^]^ our findings support a model where ORF1 functions predominantly as an intact multi‐functional protein, requiring interdomain cooperativity for HEV replication. Structural analyses revealed that the Y943A substitution disrupted critical hydrogen‐bonding networks between the α1 helix and both the α2 helix and the internal loop within the X domain. This induced a conformational shift that preferentially oriented the X domain toward Hel and RdRp, resulting in abnormally strengthened X_Y943A_ domain interactions with both Hel and RdRp. Most significantly, we identified a PC structure at the interdomain interface between X and RdRp domains. The Y943A mutation, destabilizing the PC structure, completely abolished HEV replication. This phenotype was recapitulated by targeted mutations at other essential PC‐forming residues (X: K937, R944; RdRp: G1590), definitively proving PC structural integrity to be essential for HEV replication machinery.

Using our PC‐targeted, structure‐based virtual screening platform, we identified saikosaponin D and liriopesides B as potent HEV inhibitors. Saikosaponin D and liriopesides B are two natural compounds extracted from *Bupleurum chinense* and *Ophiopogon japonicas*, respectively. Saikosaponin D is being developed as a hepatoprotective drug for the treatment of alcoholic hepatitis or hepatic fibrosis, owing to its antioxidant and anti‐inflammatory properties.^[^ ^48^, ^49^ ^]^ Similarly, liriopesides B exhibits antioxidant and anti‐aging effects.^[^ ^50^ ^]^ The pharmacological potential and favorable therapeutic window of these two compounds against HEV, combined with their well‐documented antioxidant and hepatoprotective activities, suggest they may exert a dual‐benefit effect: i) inhibiting HEV replication and ii) protecting hepatic tissue. Moreover, studies have shown that the formulation strategies, such as liposomal encapsulation, could further enhance their bioavailability.^[^ ^51^ ^]^ Therefore, these two compounds hold great potential to be further developed into anti‐HEV drugs. Collectively, our study not only validates the essential role of the PC in HEV replication, but also establishes it as a promising virus‐specific therapeutic target. Our approach aligns with the established antiviral strategies that target critical interdomain or interprotein interactions within viral replication complexes, a methodology successfully employed against multiple pathogens, including dengue virus^[^ ^52^, ^53^ ^]^ and SARS‐CoV‐2.^[^ ^54^ ^]^ Future resolution of the complete HEV ORF1 structure will be crucial to elucidate the detailed architecture of the replication complex, enabling structure‐guided design of improved antiviral agents.

*Togaviridae*, *Coronaviridae*, and *Hepeviridae* families all encode an X domain (macro domain) within their viral nonstructural polyproteins (nsPs). However, in contrast to X domain of *Togaviridae* and *Coronaviridae*, the HEV X domain shows distinctive characteristics. First, in *Togaviridae* and *Coronaviridae*, the X domain is liberated via proteolytic cleavage to function as an autonomous unit.^[^ ^19^, ^55^ ^]^ However, in HEV, existing literature and our study indicate that ORF1 polyprotein functions predominantly as an intact multi‐functional protein,^[^ ^44^, ^45^, ^47^ ^]^ and the X domain serves as an integral subunit of the ORF1 polyprotein without undergoing proteolytic processing. Second, in multiple +ssRNA viruses of *Togaviridae* and *Coronaviridae*, the ADP‐ribose hydrolase activity^[^ ^16^, ^17^, ^18^ ^]^ of X domain plays a critical role in viral replication and pathogenesis.^[^ ^19^, ^20^, ^21^, ^22^, ^55^ ^]^ However, in our study, we demonstrate that ADP‐ribose hydrolase activity of X domain per se is dispensable for HEV replication. Finally, the multiple sequence alignments reveal limited evolutionary conservation of residues forming the HEV PC among the coronavirus homologs (e.g., HCoV‐NL63, HCoV‐OC43, and SARS‐CoV‐2). Consistent with these divergences, the anti‐HEV compounds (saikosaponin D and liriopesides B) demonstrate no significant antiviral activity against HCoV‐NL63, HCoV‐OC43, and SARS‐CoV‐2 (Figures S11–S13, Supporting Information). Thus, the PC structure formed by HEV X and RdRp domains represents a unique and specific therapeutic target for developing drugs against HEV.

Through SPR analysis, we successfully characterized the binding affinities of X‐RdRp protein with saikosaponin D and liriopesides B. Building on these binding studies, we pursued structural characterization of X‐RdRp–compound complexes via X‐ray crystallography. Despite screening hundreds of crystallization conditions per compound, no co‐crystals formed. This outcome aligns with the well‐documented fact that structure determination is notoriously challenging and time‐intensive, with no guarantee of success. To date, only ≈1 00 000 unique protein structures have been resolved, representing a minute fraction (<0.01%) of the billions of known protein sequences.^[^ ^56^, ^57^, ^58^, ^59^, ^60^, ^61^, ^62^, ^63^ ^]^ This limited structural coverage reflects the substantial technical hurdles and considerable time investment (typically ranging from months to years) required per structure.^[^ ^64^ ^]^ Nevertheless, both existing literature and our findings highlight the critical need to resolve structures of the X‐RdRp protein and HEV ORF1 polyprotein—essential for discovering more viral‐specific anti‐HEV targets.

In conclusion, the X domain is an essential structural and functional component of the intact ORF1 polyprotein, which is strictly required for HEV replication. Our identification of the PC located at the interdomain interface between X and RdRp domains establishes this region as a novel, virus‐specific antiviral target. These findings provide mechanistic insights into HEV replication complex assembly and enable structure‐based development of inhibitors targeting critical interdomain interactions.

## 4 Experimental Section

### Plasmid Construction

Plasmid construction was performed using pKernow‐C1/p6‐Gluc and pKernow‐C1/p6 as backbones for reporter and non‐reporter constructs, respectively. Site‐directed mutagenesis with overlapping PCR generated: i) Gluc‐tagged mutants (N880A‐Gluc, G887V‐Gluc, A892V‐Gluc, V926A‐Gluc, P928A‐Gluc, K937A‐Gluc, L939A‐Gluc, Y943A‐Gluc, R944A‐Gluc, C947A‐Gluc, P956A‐Gluc, G961V‐Gluc, I962A‐Gluc, and G1590V‐Gluc), and ii) non‐reporter variants (N880A, G887V, A892V, V926A, P928A, L939A, and Y943A). The full‐length ORF1 polyprotein plasmid (pLVX‐ORF1‐HA) was synthesized by GENEWIZ (China), and served as the backbone for constructing its truncated variants. All primers used in this study were synthesized by GENEWIZ (China) and listed in Table S3 (Supporting Information).

### Cell Culture

HuH7 and HEK293T cells were purchased from Cell Bank of the Chinese Academy of Sciences (Shanghai, China) and cultured in Dulbecco's modified Eagle medium (DMEM, Bio‐Channel, BC‐M‐005) supplemented with 10% (v/v) fetal bovine serum (FBS, Sigma‐Aldrich, F2442), 100 µg mL^−1^ streptomycin, and 100 U mL^−1^ penicillin (Gibco, 15140122), at 37 °C in a 5% (v/v) CO_2_ humidified incubator.

### X Domains Sequence Alignment of +ssRNA Viruses

The X domain amino acid sequences from representative +ssRNA viruses, including HEV (JQ679013.1), HCoV‐229E (AF304460.1), HCoV‐NL63 (AY567487.2), TGEV (AJ271965.2), FIPV (DQ010921.1), SARS‐CoV (AY291315.1), SARS‐CoV‐2 (MN908947.3), HCoV‐OC43 (KU131570.1), MERS‐CoV (MH734114.1), BatCoV‐HKU5 (EF065509.1), MHV‐A59 (AY700211.1), IBV (M95169.1), CHIKV (MT668625.1), SINV (J02363.1), VEEV (MF590066.1), and RubV (M15240.2), were retrieved from the GenBank and aligned using MAFFT algorithm in Geneious Prime (v2023.2.1).^[^ ^65^ ^]^

### In Vitro Transcription and Viral RNA Electroporation

The pKernow‐C1/p6‐Gluc, pKernow‐C1/p6, and derived mutants were linearized with *Mlu* I (Takara, #1619). Viral RNAs were transcribed in vitro from linearized templates using the mMESSAGE mMACHINE T7 kit (Invitrogen, #1344) according to the manufacturer's protocol. Equal amounts of RNA (3 µg) were electroporated into HuH7 cells (3 × 10^6^) using an ECM630 Electro Cell Manipulator (BTX) at 270 V, 50 Ω, and 975 µF.

### Gluc Activity Assay

Secreted Gluc activity was determined using the Renilla Luciferase Assay System (Promega, #E2820).^[^ ^12^ ^]^ Briefly, 20 µL of cell culture supernatant was mixed with Renilla Luciferase Assay Reagent in a 1.5 mL luminometer tube (Corning) according to the manufacturer's protocol. Gluc activity was measured immediately using an FB 12 Luminometer (Berthold).

### Viral RNA Extraction and Quantification

Viral RNA of cell culture supernatant was extracted using the FlashPure Virus DNA/RNA kit II (GeneBetter, #R212) and reverse‐ transcribed into cDNA with the HyperScript First‐Strand cDNA Synthesis Kit (APExBIO, #K1072). Quantitative PCR was performed using 2×SYBR Green qPCR Master Mix (APExBIO, #K1070) on a LightCycler 480 II (Roche). Viral RNA copy numbers were determined from a standard curve generated with in vitro transcribed RNA and calculated as copies per milliliter of cell culture media. For intracellular viral RNA, total RNA was extracted with TRIzol Reagent (Absin, #abs9331). HEV RNA levels were quantified using the 2^−∆∆Ct^ method with β‐actin as the internal control. HEV primers: fw, TTGCCTCCGAGTTAGTCATC; rev, TGCAAAGCATTACCAGACCG. β‐actin primers: fw, AATGAGCTGCGTGTGGCT; rev, TAGCACAGCCTGGATAGCAA.

### Western Blot

Proteins in cell lysates were denatured in 3× loading buffer (375 mM Tris, 50% glycerol, 9% SDS, 9% β‐mercaptoethanol, 0.03% bromophenol blue, pH 6.8) at 95 °C for 5 min. Proteins in culture supernatant were prepared in 3× non‐denaturing loading buffer (375 mm Tris, 50% glycerol, 3% SDS, 0.03% bromophenol blue, pH 6.8) without other treatment. Proteins were separated by 10% SDS‐PAGE and transferred to 0.22 µm PVDF membranes (BIO‐RAD). After blocked with 5% skim milk for 1 h at room temperature, membranes were incubated overnight at 4 °C with either primary antibody rabbit anti‐ORF2 (in‐house, 1:4000) or mouse anti‐β‐actin (Proteintech, #66009‐1; 1:20000). Following three washes, membranes were incubated with HRP‐conjugated goat anti‐rabbit (Proteintech, #SA00001‐2; 1:5000) or anti‐mouse (Proteintech, #SA00001‐1; 1:5000) secondary antibody for 1 h at room temperature. Protein bands were visualized using a Bio‐Rad Imaging System.

### Immunofluorescence Assay (IFA)

HEV RNA‐electroporated HuH7 cells in 48‐well plates were washed twice with phosphate buffer solution (PBS) and then fixed with 4% paraformaldehyde for 30 min at room temperature. After three washes, cells were permeabilized with 0.3% Triton X‐100/PBS for 15 min, then blocked with 5% bovine serum albumin (BSA) for 60 min at room temperature. Cells were incubated overnight at 4 °C with rabbit anti‐ORF2 polyclonal antibody (in‐house, 1:3000 in PBS), followed by incubation with CoraLite594‐conjugated goat anti‐Rabbit IgG (H+L) secondary antibody (Proteintech, #SA00013‐4; 1:500) for 1 h at room temperature. Nuclei were counterstained by Hoechst (Invitrogen, #R37605; 1:500). Images were acquired using an Olympus IX51 fluorescence microscope.

### Cell Viability Assay

Cell viability was assessed using the WST‐1 Cell Proliferation and Cytotoxicity Assay Kit (KeyGen Biotech, #KGA316). Briefly, cells with or without drug treatment were incubated in 96‐well plates at 37 °C for 72 h. 10 µl WST‐1 reagent was added into each well (100 µL medium) and incubated for another 4 h at 37 °C. Absorbance at 450 nm was measured using a SYNERGY2 microplate reader (BioTek).

### Co‐Immunoprecipitation (Co‐IP) Assay

HEK293T cells co‐expressing Flag‐tagged X domain and HA‐tagged ORF1 truncations (or empty vector control) were lysed in 250 µL ice‐cold buffer (50 mm Tris, 150 mm NaCl, 0.3% N‐Dodecyl‐β‐D‐maltoside, and protease inhibitor, pH 8.0) for 30 min. Lysates were centrifugated at 13000× g for 15 min to remove cell debris. Supernatant of each sample was incubated with anti‐Flag magnetic beads (MCE, #HY‐K0207) overnight at 4 °C with rotation. Magnetic beads were washed 5× with lysis buffer, then resuspended in 50 µL loading buffer, and boiled (95 °C, 10 min). Eluted proteins were separated by 10% SDS‐PAGE, transferred to PVDF membranes, and blocked with 5% skim milk. Membranes were probed overnight at 4 °C with primary antibody anti‐Flag (Abmart, #3B9; 1:4000), anti‐HA (Abmart, #26D11; 1:4000), anti‐ORF2 (in‐house, 1:4000), or anti‐β‐actin (Proteintech, #66009‐1; 1:20000). After three washes, membranes were incubated with HRP‐conjugated secondary antibody (Proteintech, #SA00001‐1 or #SA00001‐2; 1:5000) for 1 h at room temperature. Protein bands were imaged using a Bio‐Rad Imaging system.

### Protein Purification and Surface Plasmon Resonance (SPR)

The coding sequence for the X‐RdRp protein was synthesized by GENEWIZ (China) and cloned into the pET28a expression vector, generating pET28a‐X‐RdRp with a C‐terminal hexahistidine (6×His) tag for purification. Protein expression was induced in *Escherichia coli* strain BL21 (DE3) competent cells with 0.2 mm isopropyl β‐D‐1‐thiogalactopyranoside (IPTG) at 15 °C for 16 h. The recombinant protein predominantly accumulated as inclusion bodies, which were subsequently denatured, refolded, and purified using Ni‐NTA resin. Final protein purity (>85%) was verified by 12% SDS‐PAGE.

SPR analysis was performed on a Biacore 8K instrument (Cytiva) using a CM5 sensor chip. The ligand protein (X‐RdRp) was diluted to 50 µg mL^−1^ in sodium acetate buffer (pH 4.0) and immobilized via amine coupling at a flow rate of 10 µL min^−1^. For binding assays, serial dilutions of analytes (prepared in 96‐well plates) were injected sequentially from low to high concentrations (30 µL min^−1^, 360 s contact time). Sensorgrams were processed (alignment, reference subtraction) and kinetically analyzed by global fitting to a 1:1 Langmuir binding model using Biacore Insight Evaluation Software (Cytiva, MA, USA).

### Animal Studies

All experimental procedures were approved by the Animal Care Committee of Xuzhou Medical University (IACUC No. 202504T007) and conducted in the Laboratory Animal Center (Xuzhou Medical University) under standard conditions (23 ± 1 °C, 40%–70% humidity, and 12 h light/dark cycle) with ad libitum access to food and water. Healthy male Sprague‐Dawley rats (SD, 6–8 weeks old, 200 ± 20 g) were fasted for 12 h (water allowed) before randomization into: i) vehicle control (0.5% CMC‐Na, MCE #HY‐Y0703); ii) saikosaponin D (0.5% CMC‐Na suspension, at a single dose of 400 mg kg^−1^); and iii) liriopesides B (0.5% CMC‐Na suspension, at a single dose of 400 mg kg^−1^). All were administered by oral gavage. Blood samples (0.15 mL) collected into heparinized microcentrifuge tubes at 0.17, 0.5, 1, 1.5, 2, 4, 6, 9, and 12 h post‐dosing were centrifuged for 10 min at 4000 rpm. Plasma was stored at −20 °C for UPLC‐MS/MS analysis. Pharmacokinetic parameters were determined by non‐compartmental analysis (NCA) using Phoenix WinNonlin 8.3.5 (Certara, Princeton, NJ).

### Statistical Analysis

Statistical analyses were performed using GraphPad Prism 9 software, with data presented as mean ± standard deviation (SD) from at least three independent experiments. Statistical significance was determined by Student's *t* test or one‐way analysis of variance (ANOVA), with *p* values indicated as follows: ^*^ *p *< 0.05; ^**^ *p *< 0.01; ^***^ *p* < 0.001; ns, not significant).

## Conflict of Interest

The authors declare no conflict of interest.

## Author Contributions

X.D., H.G., and W.W. were involved in the conception and design of the study. X.D. and D.Z. performed most of the experiments. X.D. analyzed the data and wrote the manuscript. D.L., Y.B., B.L., Z.L., Q.L., S.L., Y.S., J.Z., and X.L. performed some of the experiments. Q.Y. and Y.D. interpreted the data. K.Z., H.G., and W.W. reviewed and edited the manuscript. X.D., Y.S., Q.Y., Y.D., H.G., and W.W. provided funding for this study. All authors reviewed the final version of the manuscript and agreed with its content and submission.

## Supporting information



Supporting Information

## Data Availability

The data that support the findings of this study are available in the supplementary material of this article.
