# Supplementary material for: Hepatitis E Virus ORF1 Polyprotein Harbors a Pocket‐Like Cavity That Is Vital for Virus Replication and Represents a Novel Antiviral Target
Source: Adv Sci (Weinh). 2025 Oct 2;12(47):e01699. doi: 10.1002/advs.202501699 (PMC12713093; doi:10.1002/advs.202501699)
Supplement: Supplementary file 1 — Supporting Information [file ADVS-12-e01699-s001.docx]

**Hepatitis E Virus ORF1 Polyprotein Harbors a Pocket-Like Cavity That Is Vital for Virus Replication and Represents a Novel Antiviral Target**

*Xiaohui Ding**^1,2^,* *Dou Zeng^1,2^, Dan Liu^1,2^, Yingying Bian^1,2^, Bin Li^1,2^, Zheng Li^1,2^, Qiudi Li^1,2^, Shiquan Liang^1,2^, Yunlong Si^3^, Qili Yao^1,2^, Yibo Ding^1,2^, Jiahui Zhu^3^, Xiangyang Li^1,2^, Kuiyang Zheng^1,2^, Hongbo Guo^1,2*^, and Wenshi Wang^1,2*^*

^1^Department of Pathogen Biology and Immunology, School of Basic Medical Sciences, Xuzhou Medical University, Xuzhou 221004, China.

^2^Jiangsu Key Laboratory of Immunity and Metabolism, Jiangsu International Laboratory of Immunity and Metabolism, Xuzhou Medical University, Xuzhou 221004, China.

^3^Jiangsu Key Laboratory of Brain Disease and Bioinformation, Research Center for Biochemistry and Molecular Biology, Xuzhou Medical University, Xuzhou 221004, China.

*Corresponding authors. Address: No. 209 Tongshan Road, Xuzhou Medical University, Xuzhou 221004, China. E-mail addresses: hongbo.guo@xzhmu.edu.cn (H. Guo), [wenshi.wang@xzhmu.edu.cn](mailto:wenshi.wang@xzhmu.edu.cn) (W. Wang).


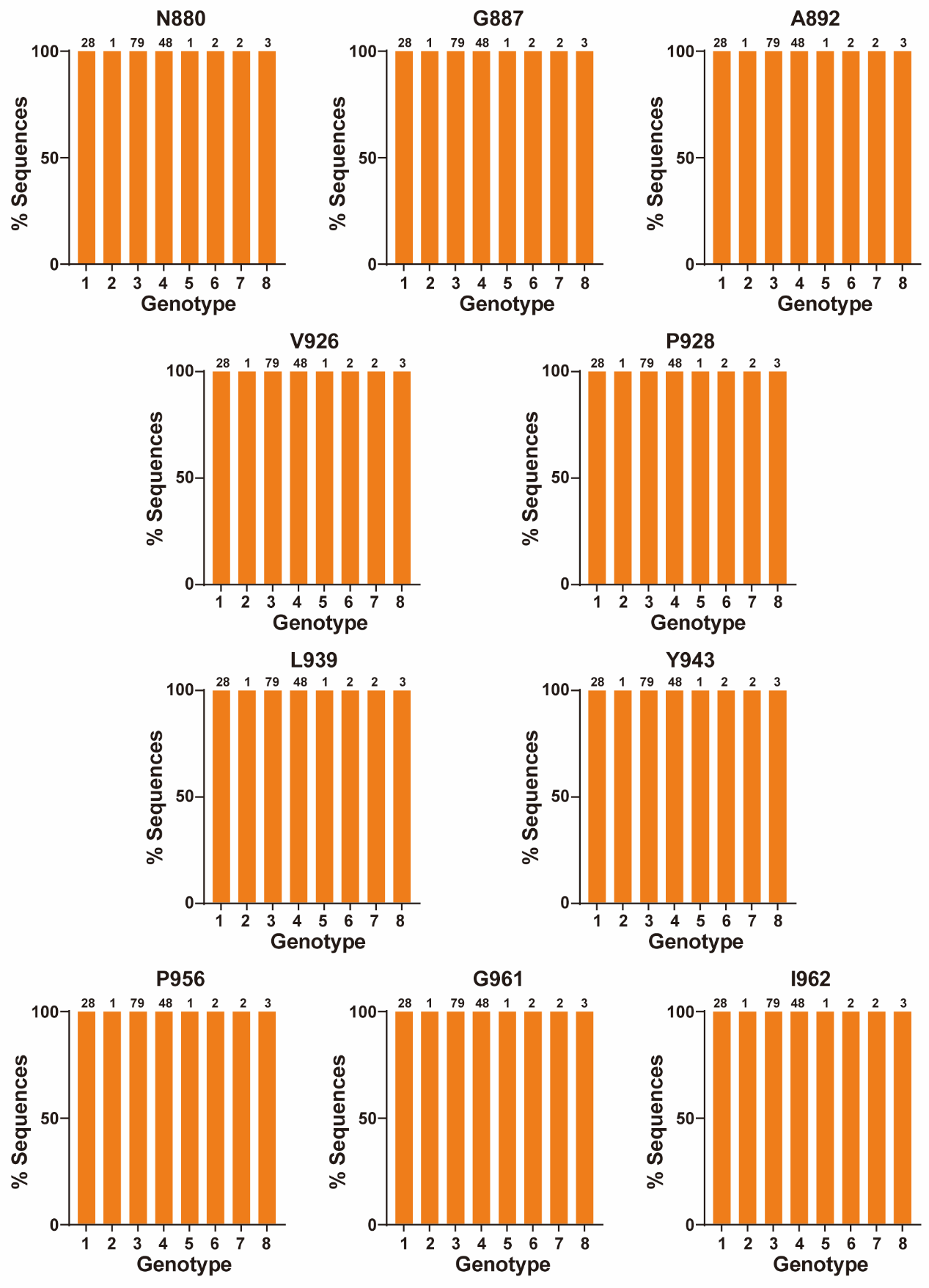


**Figure S1.** The ten residues identified in the X domain of Kernow-C1/p6 are highly conserved across all eight HEV genotypes. Full-length HEV genomes were retrieved from the GenBank database and aligned, with the following numbers of sequences analyzed per genotype: 28 for genotype 1 (AB720035, AF051830, AF076239, AF185822, AF444003, AY204877, AY230202, D10330, D11092, D11093, DQ459342, FJ457024, JF443717, JF443719, JF443720, JF443721, JF443722, JF443723, JF443724, JF443725, JF443726, JQ655734, L25595, LC061267, M73218, L08816, X98292, X99441), 1 for genotype 2 (M74506), 79 for genotype 3 (AB074918, AB074920, AB089824, AB091394, AB246676, AB291955, AB291958, AB291961, AB291962, AB291963, AB301710, AB369687, AB369689, AB369691, AB630971, AB850879, AF060668, AF060669, AP003430, EU495148, FJ653660, FJ956757, JN564006, JN837481, JQ013794, JQ013795, JQ679013, KC166971, KC618402, KF922359, KJ507955, KJ701409, AB591734, JQ026407, AB073912, AB248521, AB248522, AB481228, AB481229, AB740232, AB780453, AF082843, AF455784, AY115488, EU360977, EU375463, EU723512, EU723513, EU723514, EU723516, FJ426403, FJ426404, FJ527832, FJ998008, FJ998015, HM055578, JN906976, JQ953665, JQ953666, KF303502, KJ507956, KP698919, AB740220, AB740221, AB740222, FJ906895, FJ906896, GU937805, JQ013791, JQ013792, JQ013793, JX565469, KJ013415, AB222182, AB222183, AB222184, FJ705359, AB189071, AB236320), 48 for genotype 4 (AB602440, AB074915, AB108537, AB161717, AB197673, AB197674, AB200239, AB220974, AB291964, AB369688, AB369690, AB698654, AB909124, AB909125, AJ272108, FJ763142, HM439284, HQ634346, JQ655733, JQ655735, JQ740781, KC163335, KC492825, KC692453, LC042232, AB080575, AB481227, AY594199, AY723745, DQ279091, DQ450072, EF077630, EF570133, EU366959, EU676172, FJ610232, GU119960, GU119961, GU188851,GU206559, GU361892, HM152568, JF915746, JQ655736, JQ993308, JX855794, KF176351, KJ155502), 1 for genotype 5 (AB573435), 2 for genotype 6 (AB602441, AB856243), 2 for genotype 7 (KJ496143, KJ496144), and 3 for genotype 8 (KX387865, KX387866, KX387867).


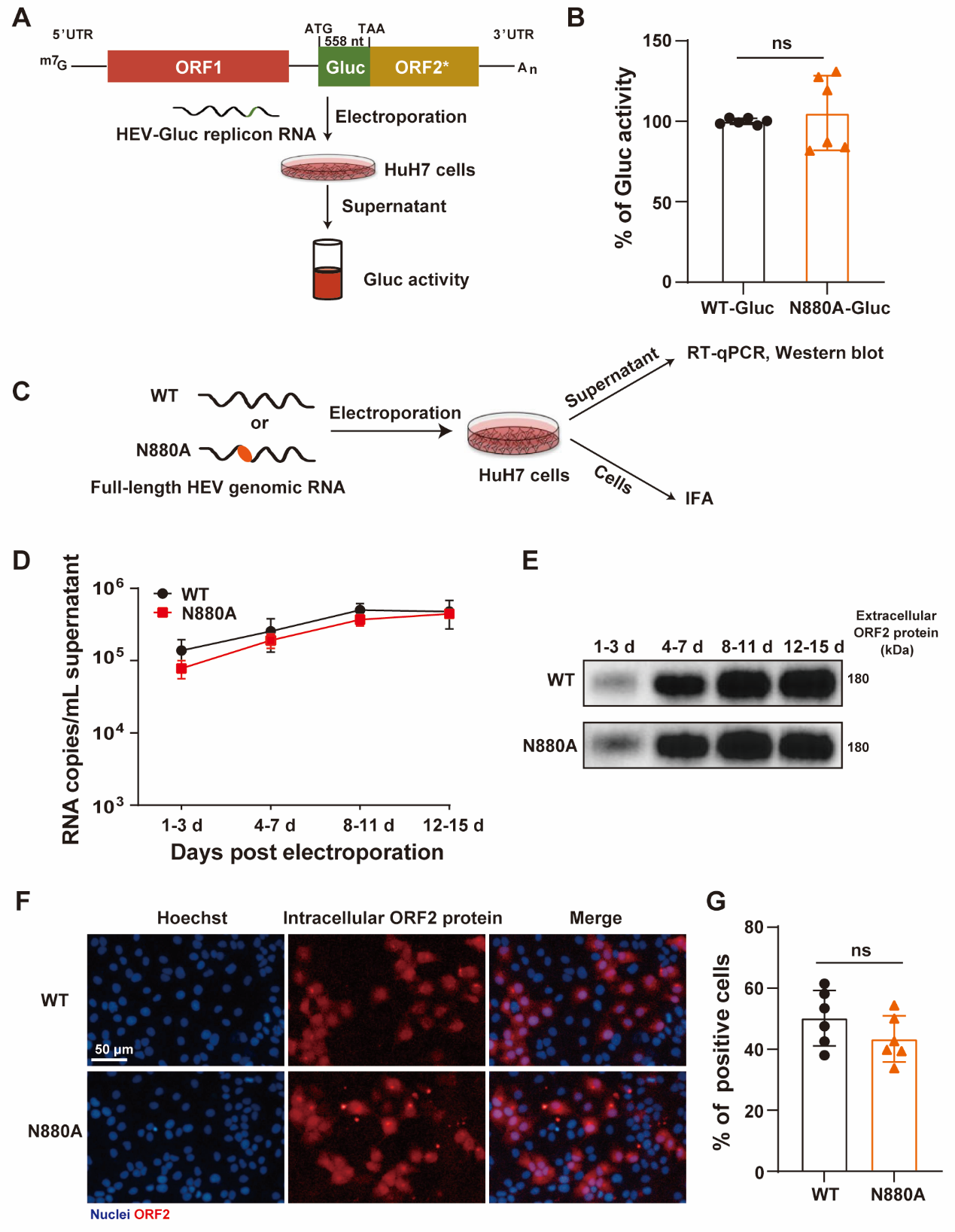


**Figure S2.** The X domain’s ADP-ribose hydrolase activity is dispensable for HEV replication. A,B) Effect of the N880A mutation on HEV replication was assessed in an HEV-Gluc replicon model (Kernow-C1/p6-Gluc). A) Schematic of the HEV-Gluc replicon assay to measure Gluc activity. B) In vitro-transcribed WT-Gluc or N880A-Gluc RNA was electroporated into HuH7 cells, and Gluc activity was quantified at 48 h post-electroporation (*n* = 3), with WT-Gluc normalized to 100%. C–G) Effect of the N880A mutation on HEV replication was assessed in a full-length HEV infectious clone model (Kernow-C1/p6). C) Schematic of the viral detection protocol measuring extracellular viral RNA levels, extracellular ORF2 protein secretion, and the percentage of ORF2-positive cells. The N880A mutation was introduced into the full-length HEV infectious clone. In vitro-transcribed WT or N880A mutant RNA was electroporated into HuH7 cells. Extracellular viral RNA levels were quantified by RT-qPCR (D). Extracellular ORF2 protein dimers (≈180 kDa) were detected by native Western blot (E). The percentage of ORF2-positive cells was quantified by immunofluorescence assay (IFA) (F,G; scale bar, 50 μm). Viral RNA levels in the supernatant (copies mL^-1^) were determined using a standard curve (*n* = 3). Data are presented as mean ± SD. ns, not significant.


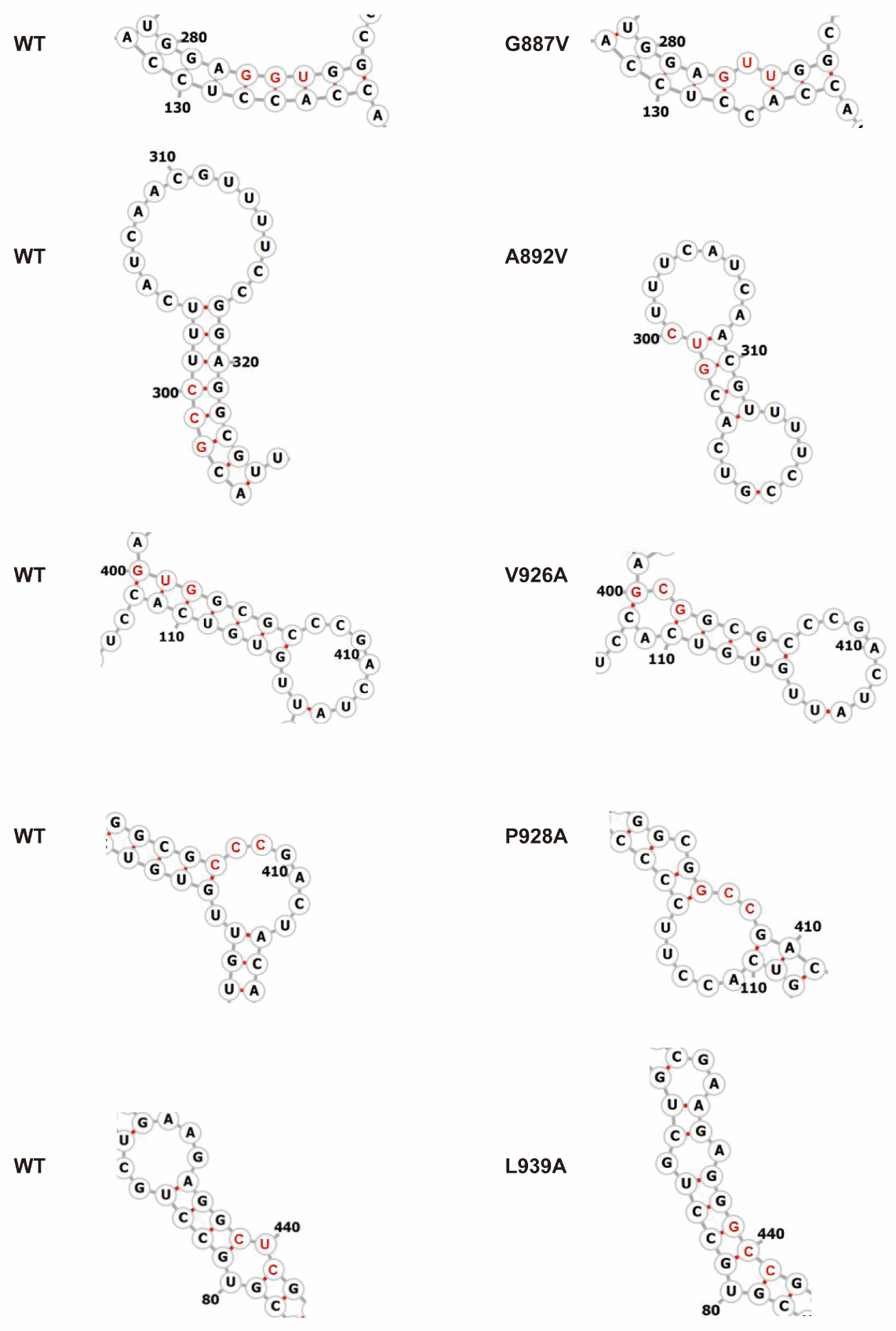


**Figure S3.** Each of the G887V, A892V, V926A, P928A, and L939A mutations introduces novel RNA loops in the X domain coding sequence. RNAalifold (http://rna.tbi.univie.ac.at/cgi-bin/RNAWebSuite/RNAalifold.cgi) was used to predict RNA secondary structure of X domain carrying each mutation.


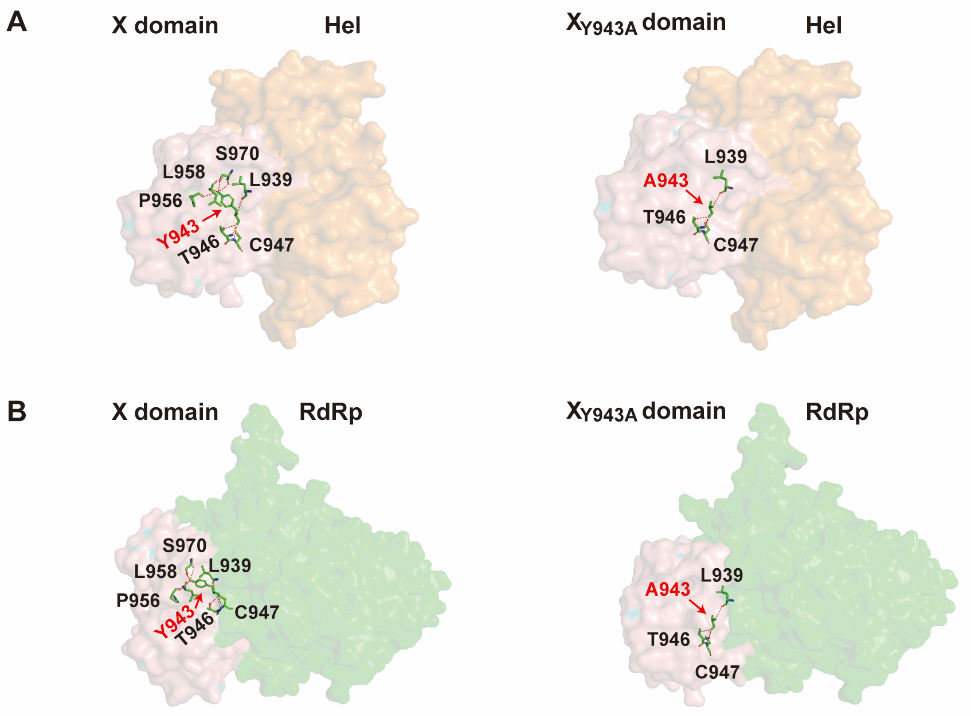


**Figure S4.** The Y943A mutation disrupts hydrogen bonds between Y943 and residues from both the α2 helix and the internal loop, thus causing X domain to tilt toward either Hel or RdRp domain. Structural models were visualized using PyMOL (v2.3.3), with red arrow indicating the Y943 or A943 position.

**
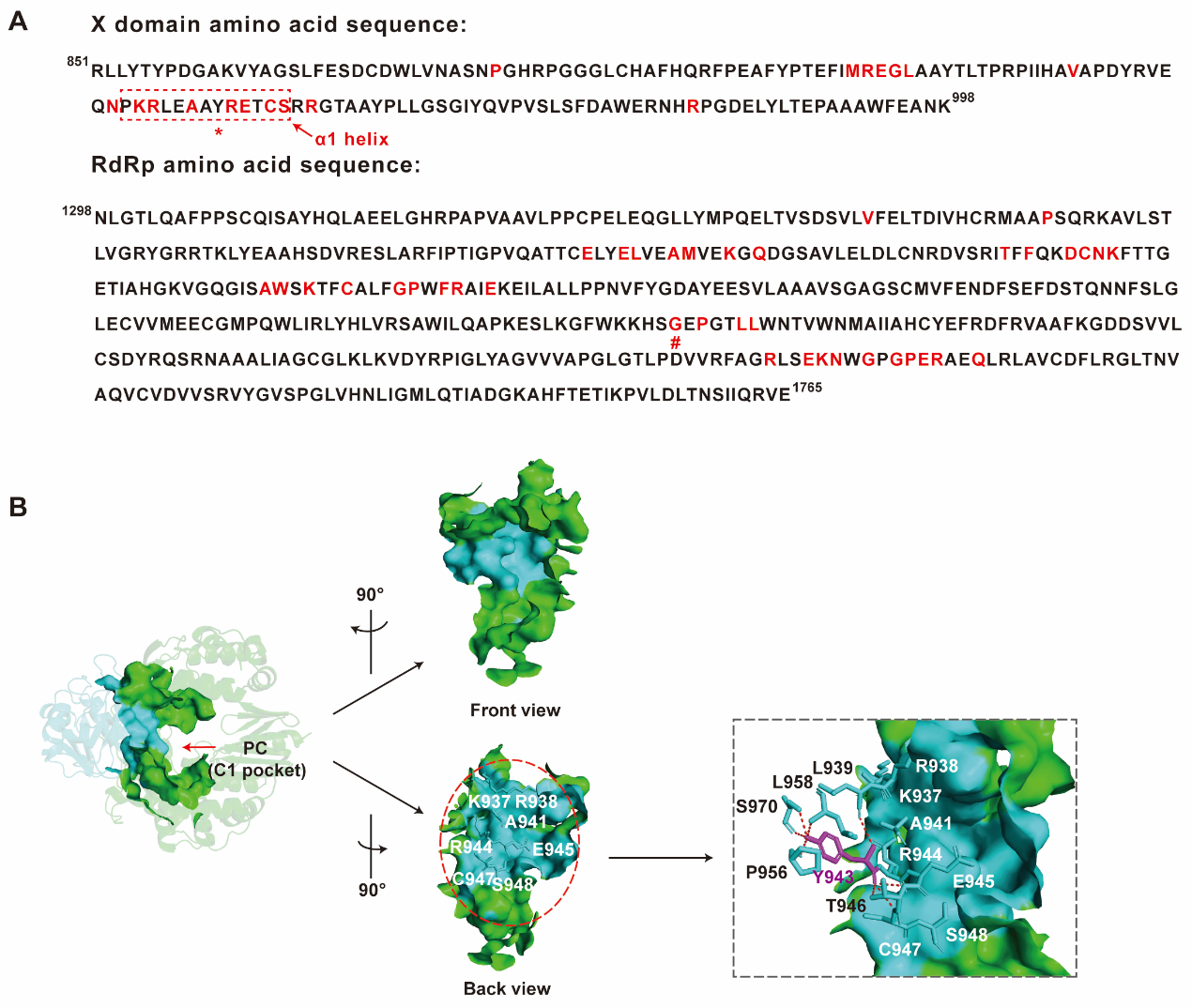
Figure S5.** Characterization of the PC structure. A) The PC was identified as the corresponding C1 pocket through CurPocket structure-based cavity detection analysis. PC-forming residues from both the X and RdRp domains are highlighted in red. B) Residues K937, R938, A941, R944, E945, C947, and S948 within X domain’s α1 helix constituted the PC base. Key residues are marked (Y943* in the X domain; G1590# in the RdRp domain). The PC structure was visualized using PyMOL (v2.3.3).


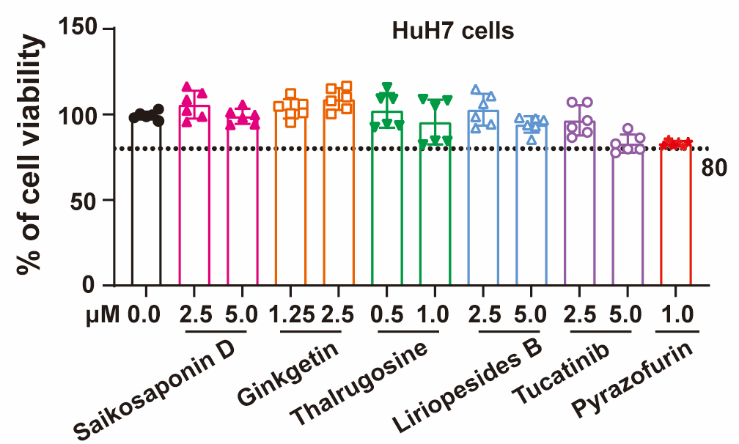


**Figure S6.** Cell viability assessment in HuH7 cells. HuH7 cells with or without drug treatment were cultured in 96-well plates for 72 h, followed by incubation with 10 μl WST-1 reagent for 4 h. Absorbance at 450 nm was measured, with the viability of untreated cells normalized to 100% (*n* = 3). Data are presented as mean ± SD.


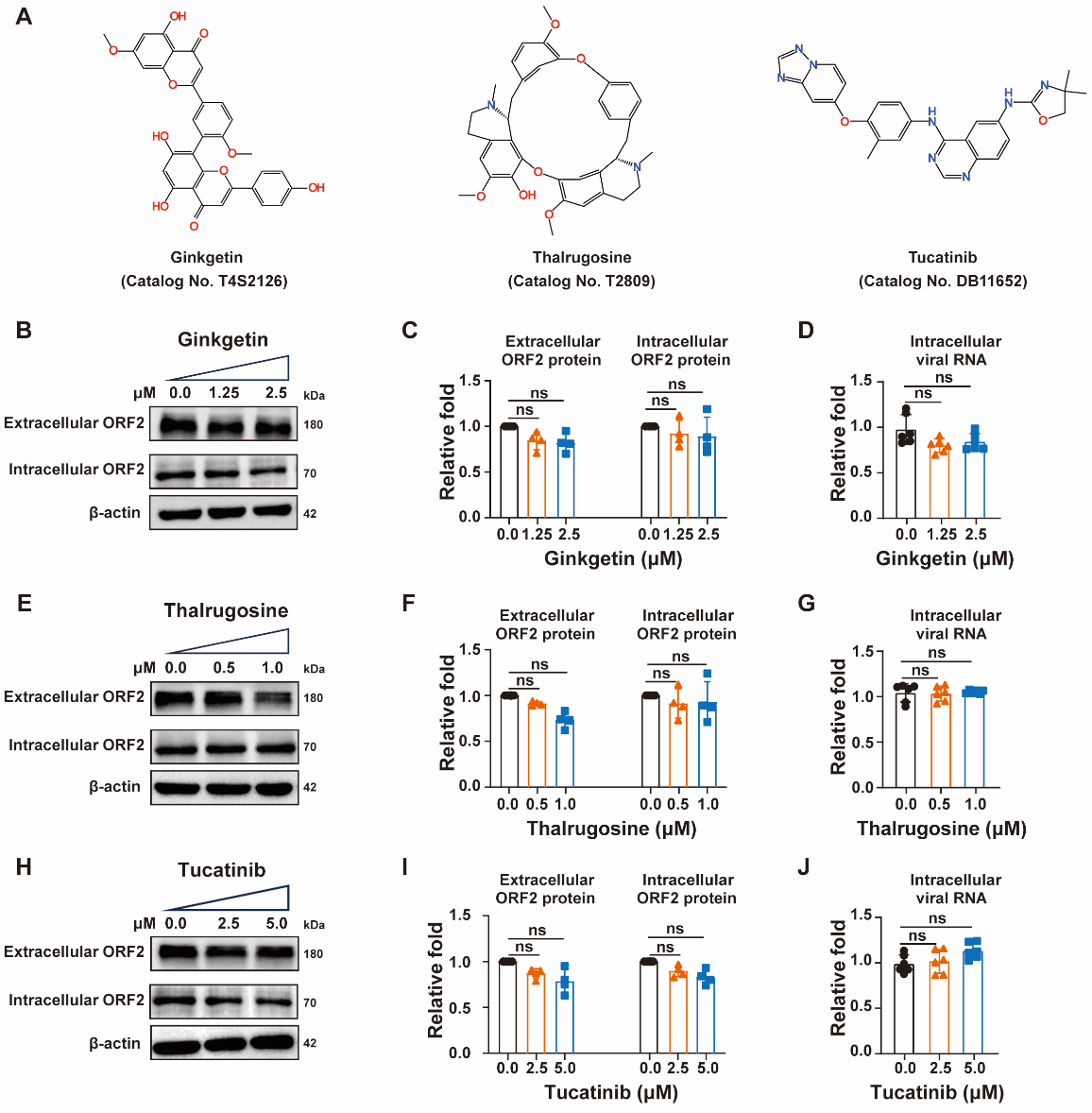


**Figure S7.** Ginkgetin, thalrugosine, and tucatinib demonstrate no significant anti-HEV activity in the full-length HEV infectious clone model. A) Chemical structures of ginkgetin, thalrugosine, and tucatinib. B–D) Antiviral assessment of ginkgetin. Extracellular and intracellular HEV ORF2 protein levels were analyzed by Western blot and quantified using ImageJ (*n* = 4). Intracellular viral RNA levels were quantified by RT-qPCR (*n* = 3). E–G) Antiviral assessment of thalrugosine. Extracellular and intracellular HEV ORF2 protein levels were analyzed by Western blot and quantified using ImageJ (*n* = 4). Intracellular HEV RNA levels were quantified by RT-qPCR (*n* = 3). H–J) Antiviral assessment of tucatinib. Extracellular and intracellular HEV ORF2 protein levels were analyzed by Western blot and quantified by ImageJ (*n* = 4). Intracellular HEV RNA levels were quantified by RT-qPCR (*n* = 3). Data are presented as mean ± SD. ns, not significant.


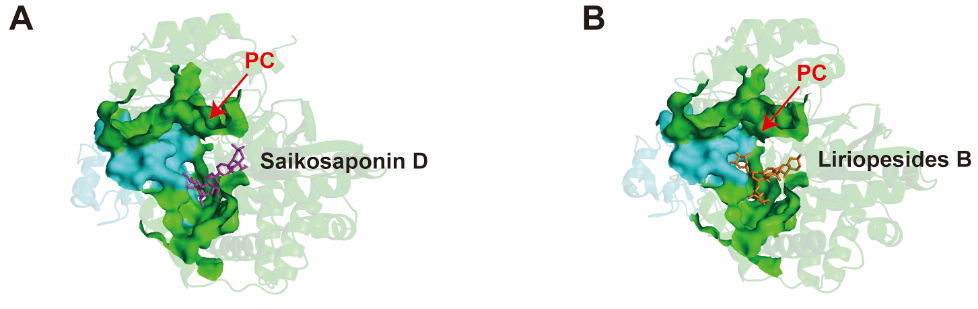


**Figure S8.** Binding modes of saikosaponin D and liriopesides B to the PC, visualized using PyMOL (v2.3.3).


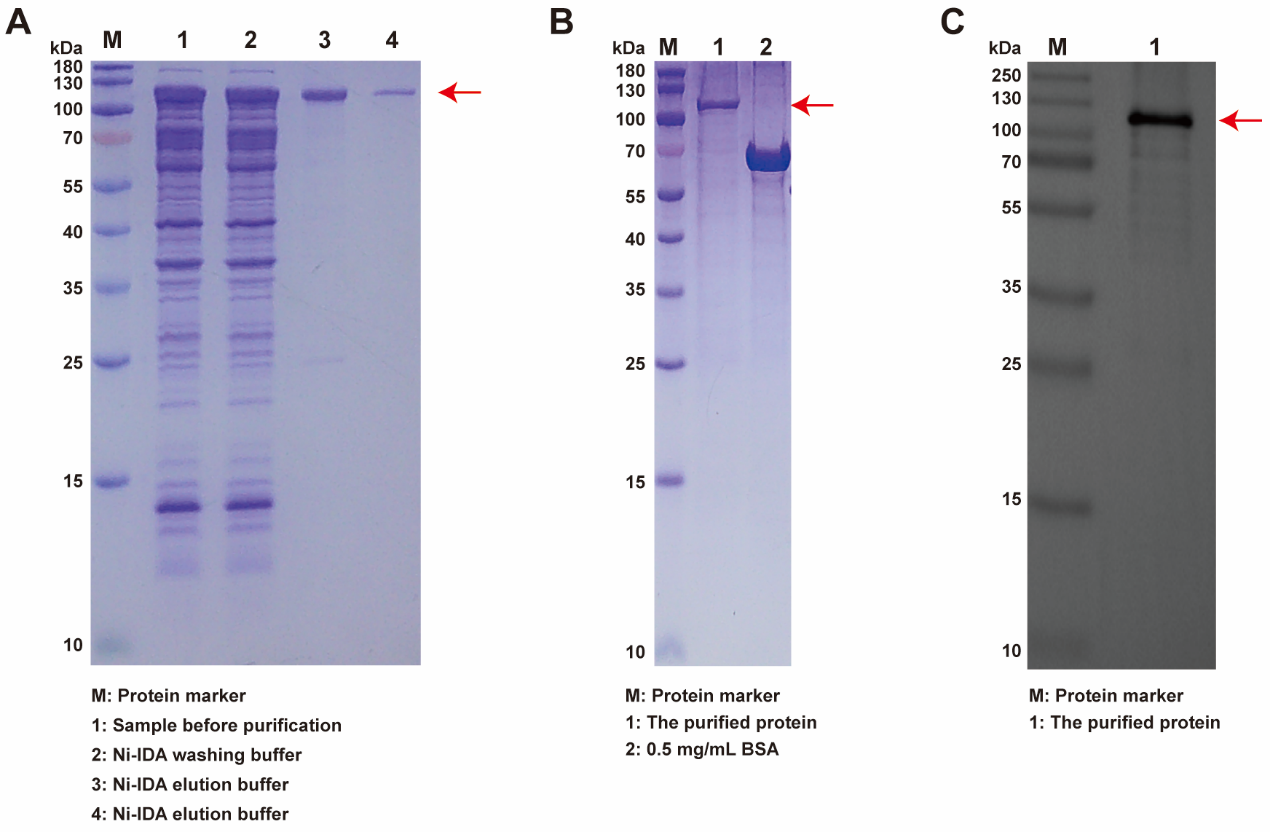


**Figure S9.** Purification and validation of the X-RdRp protein. A) Assessment of protein purification efficiency by SDS-PAGE. Lane1: Sample before purification; Lane 2: Ni-IDA washing buffer; Lane 3: Ni-IDA elution buffer; Lane 4: Ni-IDA elution buffer. B) Verification of protein purity by SDS-PAGE. Lane1: The purified X-RdRp protein; Lane 2: 0.5 mg mL^-1^ BSA. C) Validation of the X-RdRp by Western blot with His-Tag monoclonal antibody (Proteintech, #66005-1-Ig; 1:5000). Red arrow points to the X-RdRp protein band. X-RdRp, ≈107 kDa.


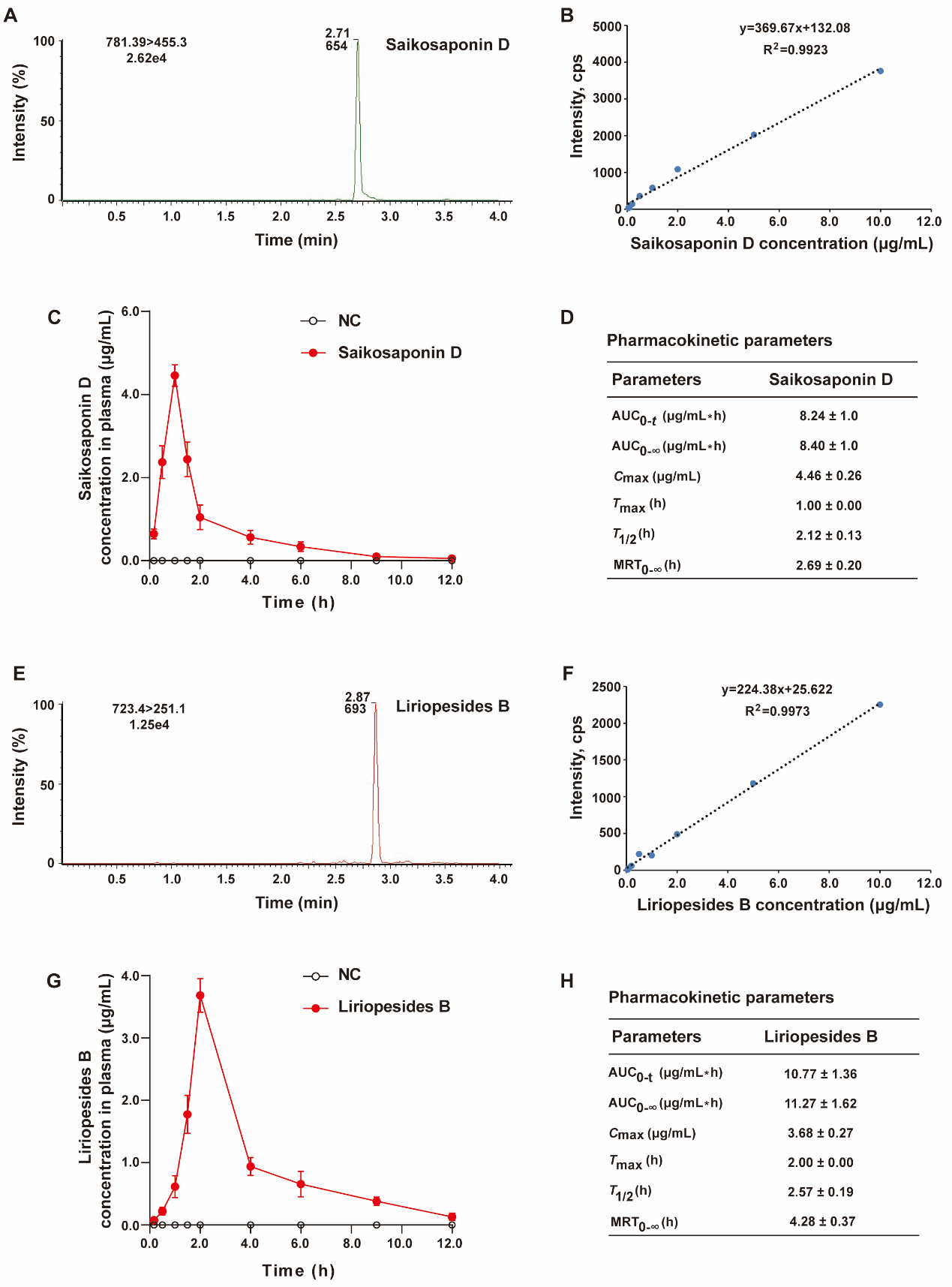


**Figure S10.** Pharmacokinetic profiling of saikosaponin D and liriopesides B in rat plasma by UPLC-MS/MS. A,B) In vitro analysis of saikosaponin D: representative UPLC chromatogram (A) and linear standard calibration curve (B). C,D) In vivo pharmacokinetics of saikosaponin D: plasma concentration-time curve (C) and pharmacokinetic parameters (D) derived using Phoenix WinNonlin 8.3.5 (*n* = 3). E,F) In vitro analysis of liriopesides B: representative UPLC chromatogram (E) and linear standard calibration curve (F). G,H) In vivo pharmacokinetics of liriopesides B: plasma concentration-time curve (G) and pharmacokinetic parameters (H) derived using Phoenix WinNonlin 8.3.5 (*n* = 3).


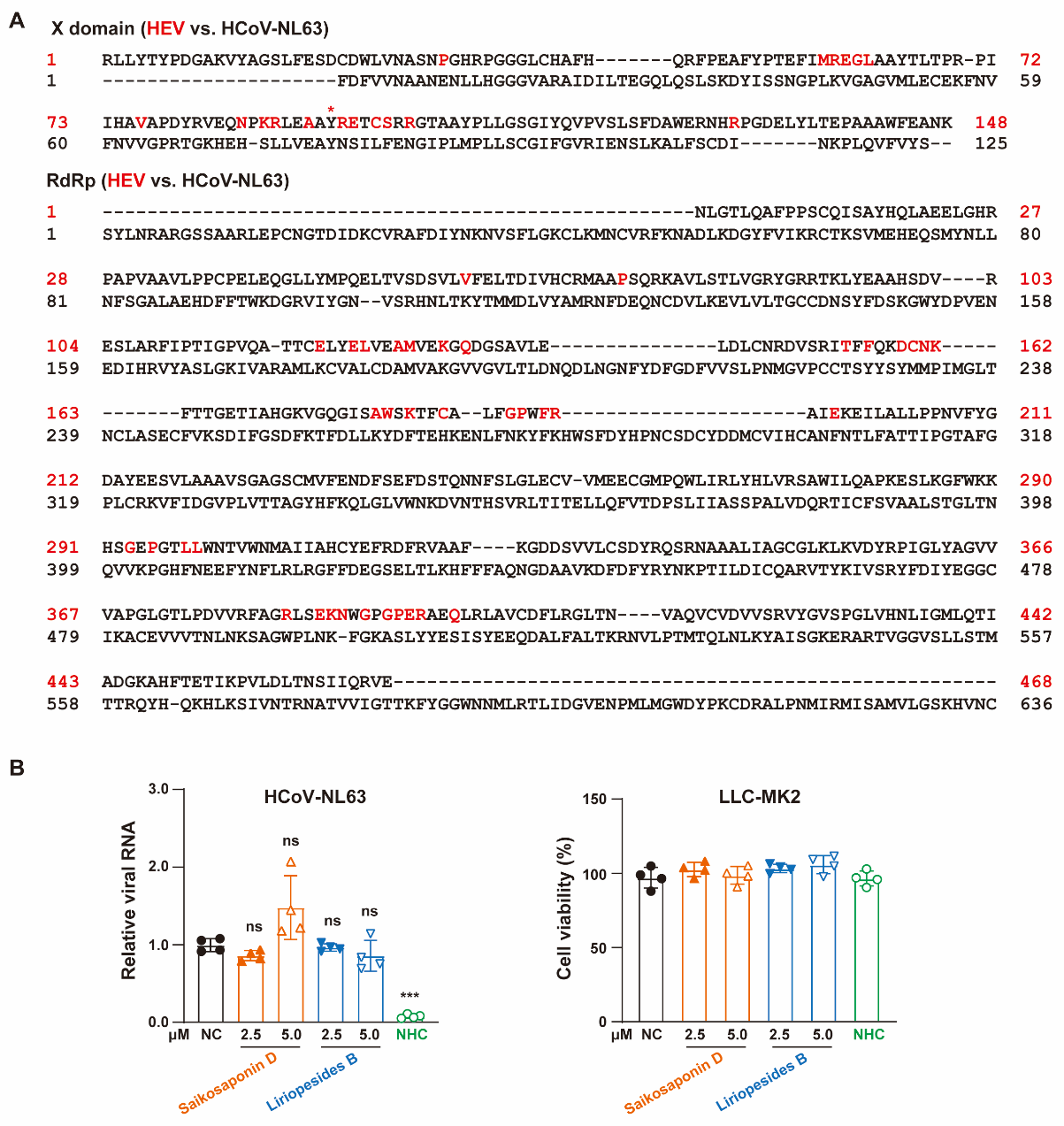


**Figure S11.** Sequence alignments of HEV and HCoV-NL63 viral proteins and evaluation of selected compounds for anti-HCoV-NL63 activity. A) Sequence alignments of the HEV X domain and RdRp with their corresponding homologs in HCoV-NL63. PC-forming residues in the HEV X domain and RdRp are highlighted in red. Y943* in the HEV X domain is marked. B) Antiviral activity and cytotoxicity profile of saikosaponin D and liriopesides B against HCoV-NL63. After 1 h of viral inoculation, LLC-MK2 cells were treated with test compounds at specified concentrations, with β-D-N4-hydroxycytidine (NHC) as a positive control.^[1]^ 48 h later, intracellular viral RNA levels were quantified by RT-qPCR and cell viability was evaluated by WST-1 assay (*n* = 4). Data are presented as mean ± SD. ***, *p*<0.001; ns, not significant.


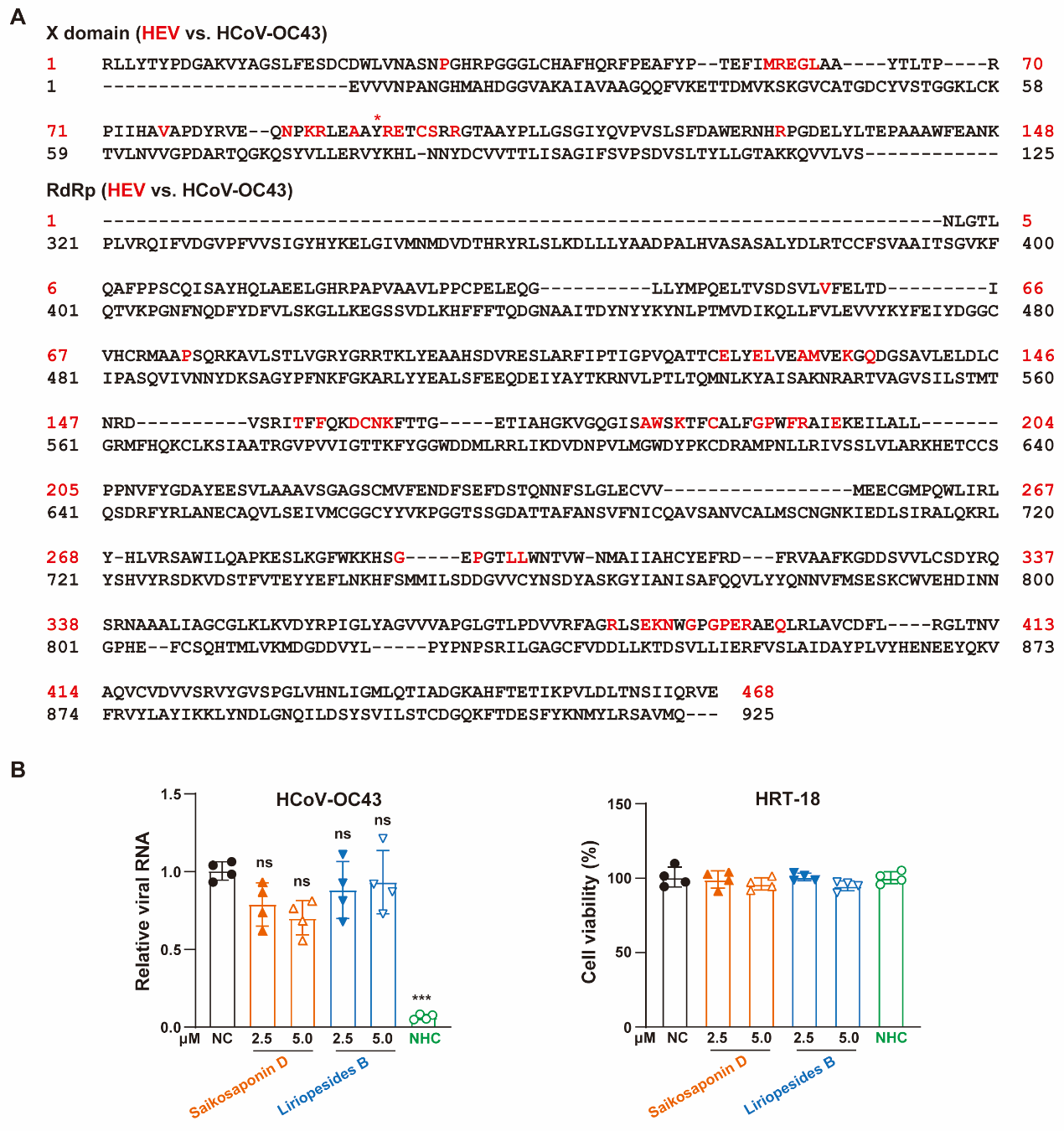


**Figure S12.** Sequence alignments of HEV and HCoV-OC43 viral proteins and evaluation of selected compounds for anti-HCoV-OC43 activity. A) Sequence alignments of the HEV X domain and RdRp with their corresponding homologs in HCoV-OC43. PC-forming residues in HEV X domain and RdRp are highlighted in red. Y943* in the HEV X domain is marked. B) Antiviral activity and cytotoxicity profile of saikosaponin D and liriopesides B against HCoV-OC43. After 1 h of viral inoculation, HRT-18 cells were treated with test compounds at specified concentrations, with NHC as a positive control.^[1]^ 48 h later, intracellular viral RNA levels were quantified by RT-qPCR and cell viability was assessed by WST-1 assay (*n* = 4). Data are presented as mean ± SD. ***, *p*<0.001; ns, not significant.


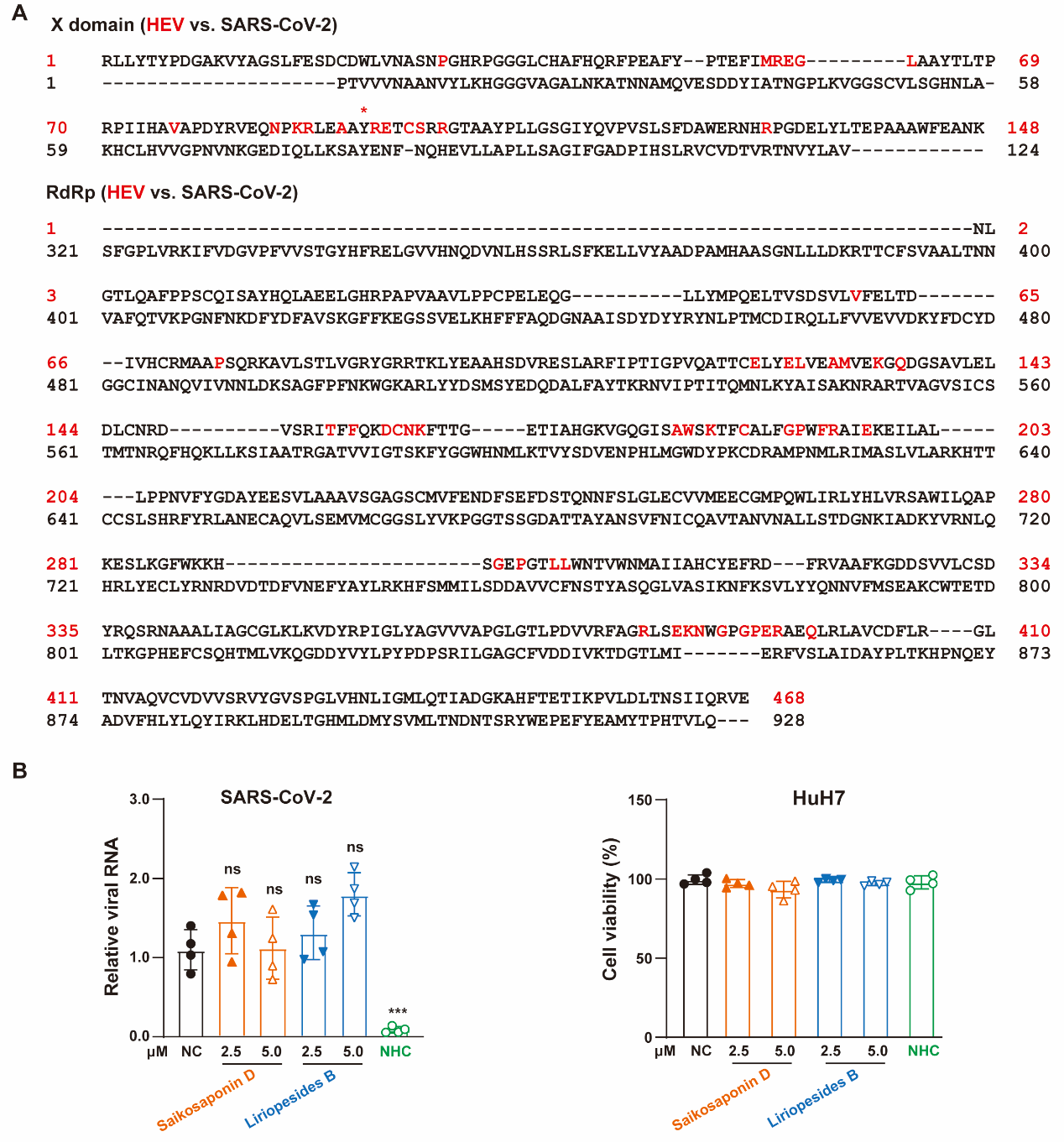


**Figure S13.** Sequence alignments of HEV and SARS-CoV-2 viral proteins and evaluation of selected compounds for anti-SARS-CoV-2 activity. A) Sequence alignments of HEV X domain and RdRp with their SARS-CoV-2 homologs. PC-forming residues in the HEV X domain and RdRp are highlighted in red. Y943* in the HEV X domain is marked. B) Antiviral activity and cytotoxicity profile of saikosaponin D and liriopesides B against SARS-CoV-2. After 1 h of viral inoculation, HuH7 cells were treated with test compounds at specified concentrations, with NHC as a positive control.^[1]^ 48 h later, intracellular viral RNA levels were quantified by RT-qPCR and cell viability was assessed by WST-1 assay (*n* = 4). Data are presented as mean ± SD. ***, *p*<0.001; ns, not significant.

Reference

[1] Dave, B., Shah, K. C., Chorawala, M. R., Shah, N., Patel, P., Patel. S., Molnupiravir: an Antiviral Drug against COVID-19, 2023, Arch. Virol., 168, 252.

| **Table S1.** 34 compounds with high predicted binding affinity to the PC selected for anti-HEV drug screening. | | | | |
| --- | --- | --- | --- | --- |
| **ID** | **Name** | **Formula** | **CAS** | **Score** |
| T4035 | Khasianine | C_39_H_63_NO_11_ | 32449-98-2 | -11.1 |
| DB11581 | Venetoclax | C_45_H_50_ClN_7_O_7_S | 1257044-40-8 | -11.0 |
| T6S0923 | Hypericin | C_30_H_16_O_8_ | 548-04-9 | -10.9 |
| DB01167 | Itraconazole | C_35_H_38_Cl_2_N_8_O_4_ | 84625-61-6 | -10.4 |
| T3S1265 | Liriopesides B | C_39_H_62_O_12_ | 87425-34-1 | -10.3 |
| DB00390 | Digoxin | C_41_H_64_O_14_ | 20830-75-5 | -10.2 |
| DB15233 | Avapritinib | C_26_H_27_FN_10_ | 1703793-34-3 | -10.2 |
| T4S0181 | Hinokiflavone | C_30_H_18_O_10_ | 19202-36-9 | -10.1 |
| DB01126 | Dutasteride | C_27_H_30_F_6_N_2_O_2_ | 164656-23-9 | -10.1 |
| DB00762 | Irinotecan | C_33_H_38_N_4_O_6_ | 97682-44-5 | -10.1 |
| T5S2129 | Sciadopitysin | C_33_H_24_O_10_ | 521-34-6 | -10.0 |
| DB01199 | Tubocurarine | C_37_H_41_N_2_O_6_ | 57-95-4 | -10.0 |
| T4S2128 | Bilobetin | C_31_H_2_0O_10_ | 521-32-4 | -9.9 |
| T4878 | Hecogenin | C_27_H_42_O_4_ | 467-55-0 | -9.9 |
| T5S0833 | Astragaloside III | C_41_H_68_O_14_ | 84687-42-3 | -9.8 |
| DB04868 | Nilotinib | C_28_H_22_F_3_N_7_O | 641571-10-0 | -9.7 |
| DB08901 | Ponatinib | C_29_H_27_F3N6O | 943319-70-8 | -9.7 |
| T6473 | Dioscin | C_45_H_72_O_16_ | 19057-60-4 | -9.6 |
| T3913 | Saikosaponin D | C_42_H_68_O_13_ | 20874-52-6 | -9.6 |
| T5S0106 | Peimisine | C_27_H_41_NO_3_ | 19773-24-1 | -9.5 |
| T2873 | Ginsenoside Rg2 | C_42_H_72_O_13_ | 52286-74-5 | -9.5 |
| T2867 | Diosgenin | C_27_H_42_O_3_ | 512-04-9 | -9.4 |
| DB00320 | Dihydroergotamine | C_33_H_37_N_5_O_5_ | 511-12-6 | -9.4 |
| DB11611 | Lifitegrast | C_29_H_24_Cl_2_N_2_O_7_S | 1025967-78-5 | -9.3 |
| T4036 | Solasodine | C_27_H_43_NO_2_ | 126-17-0 | -9.3 |
| T4S2126 | Ginkgetin | C_32_H_22_O_10_ | 481-46-9 | -9.2 |
| DB11986 | Entrectinib | C_31_H_34_F_2_N_6_O_2_ | 1108743-60-7 | -9.1 |
| DB00826 | Natamycin | C_33_H_47_NO_13_ | 7681-93-8 | -9.1 |
| T7602 | Theaflavin | C_29_H_24_O_12_ | 4670/5/7 | -9.1 |
| T3795 | Corilagin | C_27_H_22_O_18_ | 23094-69-1 | -9.1 |
| T2809 | Thalrugosine | C_37_H_40_N_2_O_6_ | 33889-68-8 | -9.1 |
| DB11231 | Lycopene | C_40_H_56_ | 502-65-8 | -9.0 |
| DB11652 | Tucatinib | C_26_H_24_N_8_O_2_ | 937263-43-9 | -9.0 |
| T0878 | Diosmin | C_28_H_32_O_15_ | 520-27-4 | -9.0 |

| **Table S2.** Dual assessment of anti-HEV activity and cytotoxicity for 34 candidate compounds. | | | |
| --- | --- | --- | --- |
| **Name** | **Concentration [μM]** | **Viral replication**  **[%]** | **Cell viability**  **[%]** |
| Ponatinib | 5 | 2.84 ± 0.41 | 25.34 ± 1.03 |
| Digoxin | 0.5 | 9.89 ± 0.18 | 28.02 ± 0.97 |
| Pyrazofurin | 1 | 45.67 ± 3.75 | 82.92 ± 1.57 |
| Hypericin | 5 | 52.31 ± 5.88 | 27.56 ± 0.55 |
| Tucatinib | 5 | 53.81 ± 3.76 | 83.00 ± 5.26 |
| Entrectinib | 5 | 59.05 ± 6.60 | 55.00 ± 3.94 |
| Corilagin | 5 | 60.99 ± 8.52 | 79.44 ± 9.22 |
| Liriopesides B | 5 | 61.14 ± 7.00 | 94.13 ± 4.77 |
| Ginkgetin | 2.5 | 61.19 ± 7.13 | 108.89 ± 6.37 |
| Saikosaponin D | 5 | 61.92 ± 7.92 | 98.93 ± 4.43 |
| Thalrugosine | 1 | 68.82 ± 7.75 | 95.51 ± 13.00 |
| Avapritinib | 5 | 72.94 ± 17.99 | 81.21 ± 7.26 |
| Hecogenin | 5 | 79.56 ± 9.24 | 94.75 ± 6.52 |
| Dioscin | 0.5 | 83.33 ± 5.49 | 102.37 ± 2.99 |
| Khasianine | 10 | 84.91 ± 3.36 | 79.90 ± 3.20 |
| Solasodine | 5 | 86.49 ± 9.48 | 96.50 ± 5.00 |
| Lycopene | 5 | 87.73 ± 6.26 | 97.37 ± 3.46 |
| Diosgenin | 5 | 87.93 ± 11.64 | 94.30 ± 4.11 |
| Itraconazole | 5 | 89.07 ± 16.75 | 36.68 ± 2.41 |
| Natamycin | 10 | 97.03 ± 11.66 | 102.42 ± 10.71 |
| Peimisine | 10 | 97.64 ± 17.29 | 108.56 ± 4.82 |
| Tubocurarine | 20 | 99.24 ± 7.99 | 112.19 ± 5.63 |
| DMSO | 0.10% | 100.00 ± 7.23 | 99.40 ± 2.31 |
| Theaflavin | 20 | 100.77 ± 1.84 | 113.70 ± 2.74 |
| Dutasteride | 10 | 100.99 ± 9.23 | 103.48 ± 3.75 |
| Doismin | 5 | 102.72 ± 8.90 | 106.46 ± 9.74 |
| Sciadopitysin | 20 | 105.16 ± 2.78 | 124.56 ± 8.19 |
| Astragaloside III | 20 | 107.68 ± 1.25 | 107.75 ± 1.07 |
| Nilotinib | 10 | 112.51 ± 17.33 | 97.63 ± 1.47 |
| Lifitegrast | 10 | 117.13 ± 10.41 | 115.05 ± 3.63 |
| Ginsenoside Rg2 | 10 | 122.28 ± 14.87 | 113.59 ± 3.07 |
| Bilobetin | 10 | 131.27 ± 27.32 | 108.32 ± 8.07 |
| Dihydroergotamine mesylate | 10 | 161.62 ± 7.04 | 101.84 ± 1.99 |
| Irinotecan | 5 | 165.15 ± 22.73 | 95.86 ± 6.29 |
| Hinokiflavone | 10 | 175.87 ± 8.04 | 101.66 ± 4.68 |
| Venetoclax | 10 | 190.46 ± 10.62 | 97.02 ± 6.16 |

| **Table S3.** Primer sequences used for plasmid construction in this study. | |
| --- | --- |
| **Name** | **Sequence** |
| Mutant-up-F | ATAAGAATGCGGCCGCCCCTGCTTCGGCTGCTGC |
| N880A-up-R | AGGGCGATGGCCCGGGGCTGAGGCGTTAACCAG |
| N880A-down-F | CTGGTTAACGCCTCAGCCCCGGGCCATCGCCCT |
| Mutant-down-R | GGAATTCCATATGTAGCAGCAACAGGTGTGGCG |
| G887V-up-R | GGCGTGACAGAGGCCAACTCCAGGGCGATGGCC |
| G887V-down-F | GGCCATCGCCCTGGAGTTGGCCTCTGTCACGCC |
| A892V-up-R | AAAACGTTGATGAAAGACGTGACAGAGGCCACCT |
| A892V-down-F | AGGTGGCCTCTGTCACGTCTTTCATCAACGTTTT |
| V926A-up-R | CCTGTAGTCGGGCGCCGCTGCGTGGATGATAGG |
| V926A-down-F | CCTATCATCCACGCAGCGGCGCCCGACTACAGG |
| P928A-up-R | CTCAACCCTGTAGTCGGCCGCCACTGCGTGGATG |
| P928A-down-F  K937A-up-R  K937A-down-F | CATCCACGCAGTGGCGGCCGACTACAGGGTTGAG  ACGCTGCCTCGAGCCTCGCCGGGTTCTGCTCAACC  GGTTGAGCAGAACCCGGCGAGGCTCGAGGCAGCGT |
| L939A-up-R | CGGTACGCTGCCTCGGCCCTCTTCGGGTTCTG |
| L939A-down-F | CAGAACCCGAAGAGGGCCGAGGCAGCGTACCG |
| Y943A-up-R | GGAGCAAGTTTCCCGGGCCGCTGCCTCGAGCCT |
| Y943A-down-F  R944A-up-R  R944A-down-F  C947A-up-R  C947A-down-F | AGGCTCGAGGCAGCGGCCCGGGAAACTTGCTCC  GACGGGAGCAAGTTTCCGCGTACGCTGCCTCGAGC  GCTCGAGGCAGCGTACGCGGAAACTTGCTCCCGTC  CGGTGCCACGACGGGAGGCAGTTTCCCGGTACGCT  AGCGTACCGGGAAACTGCCTCCCGTCGTGGCACCG |
| P956A-up-R | GCCCGAGCCTAAAAGCGCGTAAGCAGCGGTGCC |
| P956A-down-F | GGCACCGCTGCTTACGCGCTTTTAGGCTCGGGC |
| G961V-up-R | AGGGACCTGGTATATGACCGAGCCTAAAAGCGG |
| G961V-down-F | CCGCTTTTAGGCTCGGTCATATACCAGGTCCCT |
| I962A-up-R | GACAGGGACCTGGTATGCGCCCGAGCCTAAAAG |
| I962A-down-F  G1590V-up-F  G1590V-up-R  G1590V-down-F  G1590V-down-R | CTTTTAGGCTCGGGCGCATACCAGGTCCCTGTC  CCAATGCATGGTATTTGAAAATG  GAAGGGTACCGGGCTCAACAGAATGCTTCTTCCAG  CTGGAAGAAGCATTCTGTTGAGCCCGGTACCCTTC  CCGTGGTCGCGAAGTTGCTGGCCACGGC |
| Met-X-F | CCGGAATTCATGGAGGCCCATCAGTTCATCAAG |
| Met-X-R | AGCGGTTCTAGCGGTGTCCTCGGTG |
| Met-X-R-HA | CGCGGATCCTCAGGCGTAGTCGGGCACGTCGTAGGGATAAGCGGTTCTAGCGGTGTCCTCGGTG |
| X-RdRp-F | CCGGAATTCATGAGAGGCCCCGTGCGGGGGATCAGC |
| X-RdRp-R | TTCCACTCTCTGAATAATGCTGTTGGTC |
| X-RdRp-R-HA | CGCGGATCCTCAGGCGTAGTCGGGCACGTCGTAGGGATATTCCACTCTCTGAATAATGCTGTTGGTC |
| Hel-RdRp-F | CCGGAATTCATGAGCCTGGCTCTCGAGATTGACGCC |
| Hel-RdRp-R | TTCCACTCTCTGAATAATGCTGTTGGTC |
| Hel-RdRp-R-HA | CGCGGATCCTCAGGCGTAGTCGGGCACGTCGTAGGGATATTCCACTCTCTGAATAATGCTGTTGGTC |
| HVR-Hel-F | CCGGAATTCATGACCTACGAGCTGACCCCCGCCGGC |
| HVR-Hel-R | GGCCAGGAAGAAGTTGTTCACGATCAC |
| HVR-Hel-R-HA | CGCGGATCCTCAGGCGTAGTCGGGCACGTCGTAGGGATAGGCCAGGAAGAAGTTGTTCACGATCAC |
| RdRp-F | CCGGAATTCATGAATCTGGGGACCCTGCAAGCCTTC |
| RdRp-R | CGCGGATCCTCAGGCGTAGTCGGGCACGTCGTAGGGATATTCCACTCTCTGAATAATGCTGTTGGTC |
